# Acute air pollution and temperature exposure as independent and joint triggers of spontaneous preterm birth in New South Wales, Australia: a time-to-event analysis

**DOI:** 10.3389/fpubh.2023.1220797

**Published:** 2023-11-30

**Authors:** Tanya Singh, Bin Jalaludin, Shakoor Hajat, Geoffrey G. Morgan, Katrin Meissner, John Kaldor, Donna Green, Edward Jegasothy

**Affiliations:** ^1^Climate Change Research Centre, University of New South Wales, Sydney, NSW, Australia; ^2^Australian Research Council Centre of Excellence for Climate Extremes, University of New South Wales, Sydney, NSW, Australia; ^3^School of Population Health, University of New South Wales, Kensington, NSW, Australia; ^4^Ingham Institute for Applied Medical Research, University of New South Wales, Sydney, NSW, Australia; ^5^Centre on Climate Change and Planetary Health, London School of Hygiene and Tropical Medicine, London, United Kingdom; ^6^School of Public Health, Faculty of Medicine and Health, University of Sydney, Camperdown, NSW, Australia; ^7^University Centre for Rural Health, Faculty of Medicine and Health, University of Sydney, Lismore, NSW, Australia; ^8^Healthy Environments and Lives (HEAL) National Research Network, Australian National University, Canberra, ACT, Australia; ^9^Centre for Air Pollution, Energy and Health Research (CAR), Glebe, NSW, Australia; ^10^Kirby Institute, University of New South Wales, Sydney, NSW, Australia

**Keywords:** ambient temperature, air pollution, perinatal, preterm birth, survival analysis, environmental health, environmental epidemiology

## Abstract

**Introduction:**

Exposure to high ambient temperatures and air pollution has been shown to increase the risk of spontaneous preterm birth (sPTB). Less clear are the effects of cold and the joint effects of air pollution and temperature.

**Methods:**

Using a Cox proportional hazard regression model, we assessed the risk of independent and combined short-term exposure to ambient daily mean temperature and PM_2.5_ associated with sPTB in the last week before delivery on overall sPTB (weeks 23–36) and three subtypes: extremely sPTB, very sPTB, and moderate-to-late sPTB for a birth cohort of 1,318,570 births from Australia (Jan 2001–Dec 2019), while controlling for chronic exposure (i.e., throughout pregnancy except the last week before delivery) to PM_2.5_ and temperature. The temperature was modeled as a natural cubic spline, PM_2.5_ as a linear term, and the interaction effect was estimated using a multiplicative term. For short-term exposure to temperature hazard ratios reported are relative to the median temperature (18.1°C).

**Results:**

Hazard ratios at low temperature [5th percentile(11.5°C)] were 0.95 (95% CI: 0.90, 1.00), 1.08 (95% CI: 0.84, 1.4), 0.87 (95% CI: 0.71, 1.06), and 1.00 (95% CI: 0.94, 1.06) and greater for high temperature [95th percentile (24.5°C)]: 1.22 (95% CI: 1.16, 1.28), 1.27 (95% CI: 1.03, 1.57), and 1.26 (95% CI: 1.05, 1.5) and 1.05 (1.00, 1.11), respectively, for overall, extremely, very, and moderate-to-late sPTBs. While chronic exposure to PM_2.5_ had adverse effects on sPTB, short-term exposure to PM_2.5_ appeared to have a negative association with all types of sPTB, with hazard ratios ranging from 0.86 (95th CI: 0.80, 0.94) to 0.98 (95th CI: 0.97, 1.00) per 5 μg/m^3^ increase in PM_2.5_.

**Discussion:**

The risk of sPTB was found to increase following acute exposure to hot and cold ambient temperatures. Earlier sPTB subtypes seemed to be the most vulnerable. This study adds to the evidence that short-term exposure to ambient cold and heat and longer term gestational exposure to ambient PM_2.5_ are associated with an elevated risk of sPTB.

## Introduction

Exposure to both ambient temperature (low and high) and air pollution contributes to adverse birth outcomes such as low birth weight, stillbirth, and preterm birth (PTB) (1–6). Approximately 10.6% of deliveries worldwide are born preterm (7). These preterm babies are at risk of numerous health issues throughout their life course, and the families involved experience substantial economic and psychosocial burdens (8, 9). Preterm birth is defined as any live birth prior to 37 completed gestational weeks and is further categorized into extremely PTB (<28 weeks), very PTB (28–31 weeks), and moderate-to-late PTB (32–36 weeks) (10). These subcategories are important as decreasing gestational age is associated with increasing mortality, disability, intensity of neonatal care required due to complications, and, consequently, increasing expenditures (8, 11). Risk factors for each of these PTB subtypes can differ (8), and it is not clear which PTB subtypes are more susceptible to air pollution (12), temperature extremes, or their combination. Environmental factors have been linked to PTB. In addition to these, socio-economic, genetic, behavioral, infections, inflammation, prenatal complications (such as preeclampsia and gestational diabetes) and maternal factors, such as maternal age, short interpregnancy intervals, low maternal body-mass index, multiple pregnancies, and chronic conditions (such as diabetes and high blood pressure), also contribute. The precise etiology in most PTB cases, however, remains unclear (8, 9, 13).

Research on the associations between air pollution and ambient temperature with PTB highlights several issues and evidence gaps. For example, more studies focus on the effects of heat on PTB rather than on cold (6). This is despite more health issues being attributed to cold than to heat across a range of diverse settings (14). Moreover, the literature does not identify a clear period *in utero* when exposure to cold and heat (6, 15–17), or air pollution can lead to a PTB (2, 4, 5). Current evidence suggests there may be an adverse short-term effect of heat during the last gestational weeks or the week prior to delivery (1, 17). The evidence for susceptible exposure windows for adverse effects on PTB from cold and air pollution is less clear. Particulate matter ≤ 2.5 microns in aerodynamic diameter (PM_2.5_) is the most studied air pollutant due to its established causal link with cardiovascular and respiratory diseases, cancers, type 2 diabetes, and adverse birth outcomes (18, 19). For PM_2.5_, it has been suggested that exposure over the entire pregnancy, for longer periods, such as trimesters, and some specific gestational weeks is important (2, 5). Furthermore, to date, only three studies have assessed the synergistic effects of exposure to ambient air pollution and temperature (20–22). Two of these studies, one from Guangzhou, China (22), and the other one from California, USA (21), indicate that the risk of PTB to the combined exposure of air pollution and heat waves during the last gestational week before birth might be larger than the sum of the individual risks of these exposures. Such findings have important policy implications as they suggest that greater benefits could be achieved by tackling these factors simultaneously. The third study from Brisbane, Australia, conducted an effect modification analysis and found that the harmful effect of PM_2.5_ was greater at low and moderate temperatures than exposure at high temperatures (20).

New South Wales (NSW) is the most populous state in Australia. The majority of the state's population lives in a large metropolitan region with a temperate climate with hot summers and cold winters (23). Ambient PM_2.5_ levels in this region are generally low (24), although, increasingly, this population experiences weeks, or months, of hazardous levels of air pollution from wildfires (i.e., uncontrolled fires in nature areas, such as forests, grasslands, bushlands, and shrublands) and hazard reduction burns (also called controlled burns) (25, 26). Hazard reduction burns are a measure to reduce hazards from wildfires by fire management agencies (27).

It is valuable to study the independent and joint health effects of air pollution with temperature in this context, particularly as there appears to be no lower “safe” concentration level for most air pollutants (28–31) and to better understand these risks as the climate warms.

Our study examines the independent and synergistic effects of air pollution and temperature (low and high) in the week prior to birth on overall PTB and its subtypes in NSW, while accounting for “chronic” or longer term air pollution and temperature exposure, which is exposure from 1 week before delivery until conception. We chose to focus on the last gestational week before birth because previous research has highlighted the significance of heat during this period (1, 22) and the majority of studies have focused on this exposure timeframe for the interaction effect (21, 22). Additionally, we report individual effects for longer term PM_2.5_ exposure, as evidence for chronic exposure is more robust compared with other exposure durations (5).

## Methods

### Study area

The study area is the NSW Greater Metropolitan Region (GMR) which is the largest conurbation in Australia with a population of 6.2 million in 2020 (32). This region includes the cities of Sydney, Newcastle, and Wollongong, along with surrounding metropolitan areas (Supplementary Figure S1).

### Birth cohort

The study population is all singleton live births to mothers residing in the study area between 1 January 2001 and 31 December 2019 (*N* = 1,354,919). Data for these births were retrieved from the NSW Perinatal Data Collection (PDC). The PDC contains all births reported in public and private hospitals and home births within NSW. It covers information on demographic, medical, and obstetric information about the mother; information on birth date, labor onset, delivery type, and condition of the infant (33). Gestational age is measured in completed weeks, based on the best clinical estimate. Births of <20 weeks' gestation or <400 g of birth weight are not included in the register.

Relevant attributes of the births included in this study were as follows: birth date, gestational age at birth (in weeks), labor onset [spontaneous vs. non-spontaneous (i.e., induced or no labor)], parity (1, 2, ≥3), maternal age at delivery (in years), and smoking status of the mother (smoked at any time during pregnancy vs. non-smoking during the entire pregnancy).

We used each mother's residential location at the time of birth, as their geographic location, represented by their statistical area level 2 (SA2). This is a spatial unit, part of the Australian Statistical Geography Standard (ASGS) 2011, developed by the Australian Bureau of Statistics (ABS), which provides hierarchical spatial divisions for the classification of data. SA2s across Australia have an average population of ~10,000 (ranging from 3,000 to 25,000) inhabitants (34). Our study area comprises 332 SA2s. The median size of a SA2 within the GMR is 9.20 km^2^ (range: 0.86–2,189 km^2^; interquartile range: 17.00). Notably, the very large SA2s[i.e., >95th percentile(181.95 km^2^)] have a very low population density (i.e., <42 people per km^2^, which is smaller than the 5th percentile of population density) (34).

We used maternal area-level socio-economic rank as a proxy for individual socio-economic status, which is an important predictor for PTB (35, 36) and associated with adverse exposure to environmental risk factors (37). We obtained data on the Index of Relative Socio-economic Disadvantage (IRSD), which ranks each SA2 in Australia according to the relative socio-economic disadvantage to characterize socio-economic status. Within the GMR, the SA2s were grouped by quintile of IRSD score.

To limit the potential for fixed cohort bias within our study, that is, the inclusion of a higher proportion of longer gestations at the beginning and a higher proportion of PTB at the end of the study period (38), we constrained the study population to pregnancies. We focussed on pregnancies with conception dates 22 weeks before the beginning of our birth cohort (i.e., 31 July 2000) and 44 weeks prior to the end of data collection (i.e., 6 February 2019) (39). This step reduced the number of pregnancies available for our analysis to 1,327,059 (97.94%). As the survival rate of premature births before 23 weeks is extremely low, and to follow international norms (11), we only included births that occurred after 22 gestational weeks (*N* = 1,326,168, 97.88%). Finally, we restricted our analysis to those participants for whom all maternal information was available. After exclusions, which were made *a priori* and of which several were overlapping, 1,318,570 (97.32%) births from the source population were eligible for the analysis. A flowchart in Supplementary Figure S2 illustrates the different exclusions undertaken to reach the final study population.

### Air pollution and temperature data

Daily mean PM_2.5_ concentrations for each SA2 in the study region were obtained from the Centre for Air pollution, energy, and health Research Data Analysis Technology (CARDAT) platform for the study period from 1 January 2001 to 31 December 2019 (40). These data were derived from inverse distance-weighted (IWD) means of measurements from fixed-site PM_2.5_ monitors provided by the NSW Department of Planning and Environment (DPE). The SA2 means were population-weighted from estimates at a smaller spatial scale (statistical area level 1). A previous study, which used the same dataset, shows that the applied IDW method improves PM_2.5_ estimates compared with applying a mean of PM_2.5_ monitors (41).

We used the Australian Gridded Climate Data (AGCD v1.0.0) from the Australian Bureau of Meteorology (42) for the temperature data. This product provides a daily gridded dataset based on *in situ* measurements of minimum temperature (T_min_) and maximum temperature (T_max_) at 0.05° × 0.05° resolution (~5 × 5 km) (43). These grid cell temperatures were averaged over SA2 polygons. Daily average temperature (T_avg_) was calculated by taking the average of the maximum temperature of the current day and the minimum temperature of the following night. Within our study area, the AGCD dataset is based on a dense monitoring network and provides reliable estimates (43, 44). There were no missing values for the time period considered in this study. The same dataset has been used by previous health studies in NSW (41, 45).

A time series of daily average temperature and daily mean PM_2.5_ was assigned to each pregnancy based on the mother's SA2 of residence. The focus of this study was the effect of short-term acute exposure in the week prior to delivery. To do this, we calculated a 1-week exposure average for each mother, considering the week just before giving birth and starting from the day before delivery. Lag 0 for T_avg_ was in this case the same day as the T_max_ value.

### Outcome

The primary outcomes of the study were gestational age at birth and overall spontaneous preterm births (sPTB, yes/no) and subtypes of sPTB (yes/no). In the PDC, gestational age was measured in completed gestational weeks, and for each birth, labor onset was indicated (spontaneous vs. non-spontaneous). We focused on sPTB because medically initiated PTBs are emergencies without a natural onset of labor and might have different underlying mechanisms compared with sPTB (13). With non-spontaneous PTB, there is no way of knowing whether the pregnancy would have otherwise ended in a term or preterm birth. Overall sPTB was defined as a spontaneous birth between 23 and 36 gestational weeks. The sPTB subtypes, extremely, very, and moderate-to-late sPTBs were defined as spontaneous births between 23 and 27 completed gestational weeks, 28 and 31 completed gestational weeks, and 32 and 36 completed gestational weeks, respectively.

### Statistical analysis

Cox proportional hazard regression models were fitted to explore the associations between PM_2.5_ and T_avg_, independently and synergistically, and overall sPTB, and its subtypes, during the last gestational week at risk, by treating sPTB as a time-to-event outcome. First, we assessed the individual effects of mean PM_2.5_ and T_avg_ in the last gestational week before delivery on sPTB after adjusting for all available confounders. We then assessed the potential synergistic effects of these two exposures on a multiplicative scale, by introducing a product term between the two variables in the model, while considering the same confounders as in the first step. We assessed the hazard for overall sPTB (model *N* = 1,318,570), and each sPTB subtype, leading to four different models. For extremely sPTB, we censored all births at week 28 (model *N* = 1,318,570). For very sPTB, we excluded all births <28 gestational weeks and censored births at week 32 (model *N* = 1,315,447). For moderate-to-late sPTB, we excluded all births prior to week 32 (model *N* = 1,309,274). Finally, for overall sPTB and moderate-to-late sPTB, all term births were censored at week 37, when they were not at risk for PTB anymore. In all models, non-spontaneous PTB [*n* = 32,295 (2.45%)] was considered “at risk” until birth and censored after birth (i.e., cause-specific proportional hazard model).

Covariates included in this study were selected *a priori* as potential confounders [see directed acyclic graph DAGitty (46); Supplementary Figure S3]. We controlled for maternal age, smoking status during pregnancy, parity, mother's SA2 IRSD ranked as quintiles, seasonality (month of conception), longer term trends (year of conception), and whether the birth took place during a weekday vs. weekend or public holiday. Because relatively younger and older mothers have been associated with adverse birth outcomes, we used a natural cubic spline with three degrees of freedom (df) to model a non-linear relationship with maternal age (20, 36, 47). To control for any long-term time trends, we included a natural cubic spline with two degrees of freedom for the year of conception (20, 36). Finally, to isolate the short-term effect of PM_2.5_ and temperature, we controlled for “chronic” exposure to PM_2.5_ and T_avg_, which is exposure from 1 week before delivery until conception (hereafter termed longer term gestational exposure) (22). Pregnancy complications, such as preeclampsia and gestational diabetes, were not included as covariates in the models because they can be on the causal pathway of antenatal exposure to PM_2.5_ and temperature and birth outcomes (22, 48, 49).

The impact of short-term and longer term gestational PM_2.5_ on PTB was considered linear based on previous literature (4), with the hazard ratios (HRs) and 95% confidence intervals (CIs) calculated per 5-μg/m^3^ increase in PM_2.5_ concentration. Associations between ambient temperature and health outcomes often take a non-linear (a *J*- or *U*-shaped curve) exposure–response relationship (50). Therefore, both short-term and longer term gestational exposure to T_avg_ were included as natural cubic splines with three degrees of freedom. In this way, the potential increased risk at low and high temperatures could be considered. For temperature, we estimated HRs for sPTB at high (95th percentile, i.e., hot) and low (5th percentile, i.e., cold) temperatures, relative to the median of the weekly mean temperature (51). For the interaction effect of temperature with PM_2.5_ at low and high values (5th percentile and 95th percentile), the same median reference temperature as for the independent effect was used. The moderating effects of low and high temperatures on PM_2.5_ on the other hand were compared with the effects at the lowest mean weekly value of PM_2.5_.

To check for multicollinearity, correlations between the different exposure variables were estimated using Pearson's correlation.

We performed two sensitivity analyses to assess the robustness of our results. To check whether our results were sensitive to the selected short-term exposure window of 1 week and to make them more comparable with other studies that looked at longer exposure windows [e.g., (51, 52)], we chose a period of 4 weeks before birth (hereafter termed 4-week PM_2.5_ and 4-week T_avg_). The longer term gestational exposure to PM_2.5_ and T_avg_ was adjusted accordingly (i.e., from 4 weeks before birth until the conception date). As the shape of the exposure–response function of the association of PM_2.5_ with PTB is unknown (53) and could be non-linear, especially at high PM_2.5_ concentration levels (54), we performed a sensitivity analysis in which the form of PM_2.5_ for the short-term exposure was non-linear by fitting a natural cubic spline with three degrees of freedom.

All analyses were performed in R (version 1.4.1717; R Development Core Team), and we used the “survival” (55), “smoothHR” (56), “splines” (57), and “visreg” (58) packages.

This research project was approved by the University of New South Wales Human Research Low Risk Ethics Advisory Committee Panel E, Reference Number: HC200817.

## Results

### Spontaneous preterm births

Among the 1,318,570 births included in our study population, 38,900 (2.95%) were overall sPTB, 2,001 (0.15%) were extremely sPTB, 3,059 (0.23%) were very sPTB, and 33,840 (2.57%) were moderate-to-late sPTB (Table 1). Mothers who were younger, smoked at any time during pregnancy, and first-time and at least third-time mothers were more likely to have an sPTB compared with all births. The risk of sPTB increased with increasing area-level socio-economic disadvantage of the mother.

**Table 1:** Description of the study population (i.e., all mothers who gave a live birth within the Greater Metropolitan Region of New South Wales, Australia, between 1 January 2001 and 31 December 2019) by maternal characteristics and birth types.

**Maternal characteristics**		**Overall sPTB (*n*= 38,900)**	**Extremely sPTB (*n*= 2,001)**	**Very sPTB (*n*= 3,059)**	**Moderate-to-late sPTB (*n*= 33,840)**	**All births (*n*=1,318,570)**
Gestational age {weeks [mean (SD)]}		34.1 (2.8)	25.2 (1.4)	29.8 (1.1)	35 (1.2)	39.0 (1.7)
Maternal age {years [mean (SD)]}		30.2 (5.8)	30.0 (6.3)	30.2 (6.1)	30.2 (5.7)	30.6 (5.4)
Maternal age categorized [years, *n* (%)]	< 20	1,567 (4.0)	125 (6.2)	162 (5.3)	1,280 (3.8)	30,899 (2.3)
	between 20 and 34	28,166 (72.4)	1,366 (68.3)	2,152 (70.3)	24,648 (72.8)	966,676 (73.3)
	>34	9,167 (23.6)	510 (25.5)	745 (24.4)	7,912 (23.4)	320,995 (24.3)
Smoking status [*n* (%)]	Yes	6,603 (17.0)	373 (18.6)	617 (20.2)	5,613 (16.6)	118,656 (9.0)
Area socio-economic status in quintiles [*n* (%)]	Most disadvantaged	10,838 (27.9)	610 (30.5)	957 (31.3)	9,271 (27.4)	326,232 (24.7)
	Second	8,007 (20.6)	424 (21.2)	636 (20.8)	6,947 (20.5)	256,920 (19.5)
	Middle	6,953 (17.9)	359 (17.9)	520 (17.0)	6,074 (17.9)	239,647 (18.2)
	Fourth	6,677 (17.2)	312 (15.6)	481 (15.7)	5,884 (17.4)	244,922 (18.6)
	Least disadvantaged	6,425 (16.5)	296 (14.8)	465 (15.2)	5,664 (16.7)	250,849 (19.0)
Parity [*n* (%)]	1	19,051 (49.0)	1,049 (52.4)	1,522 (49.8)	16,480 (48.7)	581,146 (44.1)
	2	11,289 (29.0)	527 (26.3)	855 (28.0)	9,907 (29.3)	452,611 (34.3)
	= >3	8,560 (22.0)	425 (21.2)	682 (22.3)	7,453 (22.0)	284,813 (21.6)

sPTB, spontaneous preterm birth; SD, standard deviation; *n*, number. All births include live spontaneous preterm births, all live non-spontaneous births, and live term births (i.e., gestational age > 36 weeks). It should be noted that this table does not represent the comparisons made in each of the Cox proportional hazard models. Term and non-spontaneous births together, however, represent most of all births in our dataset and therefore are a good approximation for the comparisons used in each model.

### Ambient PM_2.5_ and ambient temperature exposure

Mean PM_2.5_ in the week before delivery was similar across sPTB subtypes, including overall spontaneous sPTB (Table 2). The same was the case for longer term exposure to PM_2.5_, noting that all sPTB groups had a slightly lower mean and median exposure than all births. Mean T_avg_ exposure the week before birth and long-term gestational exposure were also very similar across all births and sPTB groups.

**Table 2:** Exposure to PM_2.5_ (μg/m^3^) and T_avg_ (°C) in the week prior to delivery and longer term gestational exposure (i.e., from conception till the week prior to delivery) by birth type for all live births in the Greater Metropolitan Region of New South Wales between 1 January 2001 and 31 December 2019.

**Exposure type**	**Birth type**	**Mean**	**Standard deviation**	**Minimum**	**25th**	**Median**	**75th**	**Maximum**
PM_2.5_ last gestational week (μg/m^3^)	Overall sPTB	7.29	3.39	0.83	5.38	6.80	8.48	83.95
	Extremely sPTB	7.17	3.01	1.84	5.30	6.72	8.39	40.67
	Very sPTB	7.30	3.64	0.83	5.36	6.74	8.38	62.54
	Moderate-to-late sPTB	7.36	3.76	1.02	5.36	6.78	8.47	83.95
	All births	7.33	3.38	0.82	5.45	6.83	8.53	83.95
T_avg_ last gestational week (*°C*)	Overall sPTB	17.91	4.28	3.97	14.04	18.08	21.54	30.18
	Extremely sPTB	18.16	4.30	7.26	14.26	18.55	21.70	28.64
	Very sPTB	18.03	4.32	8.68	14.10	18.35	21.71	30.18
	Moderate-to-late sPTB	17.97	4.30	3.97	14.06	18.22	21.62	29.83
	All births	17.97	4.29	3.97	14.10	18.14	21.59	31.05
PM_2.5_ longer term gestational (μg/m^3^)	Overall sPTB	7.28	1.31	3.51	6.32	7.31	8.09	12.75
	Extremely sPTB	7.26	1.47	2.48	6.25	7.15	8.14	14.47
	Very sPTB	7.26	1.38	3.54	6.26	7.23	8.12	12.93
	Moderate-to-late sPTB	7.21	1.32	2.50	6.25	7.21	8.04	13.07
	All births	7.30	1.27	3.51	6.36	7.38	8.09	12.75
T_avg_ longer term gestational (*°C*)	Overall sPTB	18.10	1.95	9.41	16.43	18.07	19.79	23.52
	Extremely sPTB	17.98	2.87	9.41	15.26	17.88	20.70	23.52
	Very sPTB	18.16	2.36	9.96	16.00	18.18	20.33	22.81
	Moderate-to-late sPTB	18.11	1.89	10.21	16.46	18.04	19.75	23.02
	All births	18.10	1.75	9.41	16.59	18.03	19.64	23.60

sPTB, spontaneous preterm birth; 25th, 25th percentile value; 75th, 75th percentile value. Overall sPTB (*n*)= 38,900, extremely sPTB (*n*) = 2,001, very PTB (*n*) = 3,059, moderate-to-late PTB (*n*) = 33,840, all births (*n*) = 1,318,570. All births include all live spontaneous preterm births, live non-spontaneous births, and live term births (i.e., gestational age > 36 weeks). This table does not represent the comparisons made in each of the Cox proportional hazard models. Term and non-spontaneous births, however, represent the vast majority of all births in our dataset and therefore are a good approximation for the comparisons used in each model.

Exposure to PM_2.5_ in the last gestational week before birth was slightly higher during winter (June–August) and summer (December–February) for all births, including sPTB, than in spring and autumn (Supplementary Table S1, see median and mean values). Pregnant women living in the inner suburbs of Sydney had on average the highest exposure to PM_2.5_ (Supplementary Figure S4), and mothers living in SA2s closer to the coast were exposed to higher temperatures during the last week of their pregnancies (Supplementary Figure S5).

There was no correlation between short-term PM_2.5_ and short-term T_avg_ exposure (*r* = 0.08). PM_2.5_ exposure the week before birth correlated weakly with longer term gestational PM_2.5_ exposure (*r* = 0.26), and T_avg_ the week before birth had a moderate negative correlation with longer term gestational T_avg_ (*r* = −0.43) (Supplementary Table S2).

### Ambient PM_2.5_ and risk of sPTB

**Figure 1:**
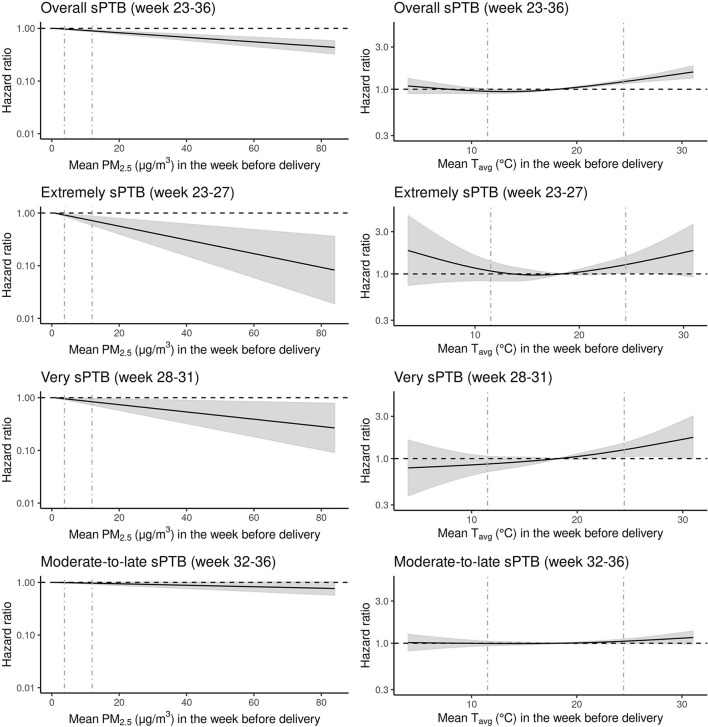
Hazard ratios (HRs) of spontaneous preterm birth (sPTB) associated with exposure to mean particulate matter equal to or <2.5 microns [PM_2.5_ (μg/m^3^)] and mean temperature T_avg_ (C°) the week before delivery for all live births (N = 1,318,570) in the Greater Metropolitan Region of New South Wales between 1 January 2001 and 31 December 2019 are shown with the black line. Gray areas around the black hazard ratio lines show 95% confidence intervals (CI). The vertical gray dashed lines represent the 5th and 95th percentile marks. The relationship between PM_2.5_ and sPTB was modeled as linear and for T_avg_ as a natural cubic spline with three degrees for freedom and median temperature (18.1 C°) as the reference point. All models were adjusted for year and month of conception, weekdays vs. weekends/holidays, maternal age, parity, smoking during pregnancy, area-level socio-economic status of the mother and exposure to longer term gestational T_avg_ and PM_2.5_ (i.e., exposure across the entire pregnancy except for the last week before delivery).

The relationship of longer term gestational exposure to PM_2.5_ with all sPTB groups was hazardous for increasing levels of PM_2.5_ (Supplementary Table S3). The HRs were 1.07 (95% CI: 1.02–1.12), 1.34 (95% CI: 1.10–1.64), 1.2 (95% CI: 1.02–1.43), 1.00 (95% CI: 0.95–1.06) per 5 μg/m^3^ increase in longer term gestational PM_2.5_ for overall, extremely, very, and moderate-to-late sPTBs, respectively. Short-term exposure to PM_2._5, on the other hand, was associated negatively with overall and all sPTB subtypes (Figure 1; Supplementary Table S3). The HRs were 0.95 (95% CI: 0.94–0.97), 0.86 (95% CI: 0.79–0.94), 0.92 (95% CI: 0.87–0.99), 0.98 (95% CI: 0.97–1.00) per 5 μg/m^3^ increase in PM_2.5_ for overall, extremely, very, and moderate-to-late sPTBs, respectively (Supplementary Table S3).

### Ambient temperature and risk of sPTB

The relationship between overall, very, and moderate-to-late sPTBs and short-term exposure to T_avg_ indicated a J-shaped form (Figure 1; Supplementary Table S4). However, the exposure–response curve for very sPTB seemed almost linear. For extremely sPTB, the exposure–response relationship was *U*-shaped. The HR for overall sPTB at a lower temperature [5th percentile (11.5°C)] was 0.95 (95% CI: 0.90, 1.00) compared with the median temperature (18.14°C). For higher temperature [95th percentile (24.5°C)], the risk of sPTB increased to 1.22 (95% CI: 1.16, 1.29) compared with the reference. For extremely sPTB, the HR at low temperature was 1.08 (95% CI: 0.084, 1.39), and at high temperature was 1.27 (95% CI: 1.03, 1.57). For very sPTB, the HR was 0.87 (95% CI: 0.71, 1.06) for low and 1.26 (95% CI: 1.05, 1.50) for high temperatures. Finally, for the moderate-to-late sPTB subtype, the risk of sPTB was 1.00 (95% CI: 0.94, 1.06) and 1.14 (1.06, 1.2) at low- and high-temperature values, respectively, compared with the median temperature.

### Two-way interaction between temperature and PM_2.5_ on risk of sPTB

There was an indication for increasing HRs (effect estimates converged toward 1.00) for the association between short-term PM_2.5_ and all sPTB groups as short-term mean temperature (T_avg_) increased from the 5th (11.5°C) to the 95th (24.5°C) percentile (Table 3; Supplementary Figure S6). Apart from this pattern, temperature did not influence the association between short-term PM_2.5_ and sPTB, except for moderate-to-late sPTB. In all other cases, the confidence intervals contained the point estimate from the comparison group.

**Table 3:** Spontaneous preterm (sPTB) birth hazard ratios for the interaction effect between mean PM_2.5_ and mean T_avg_ in the week before delivery for all live births in the Greater Metropolitan Region of New South Wales between 1 January 2001 and 31 December 2019.

		**PM**_**2.5**_ **percentile**			
**sPTB group**	**Temperature percentiles**	**5th (3.7** μ**g/m**^3^**)**	**25th (5.5** μ**g/m**^3^**)**	**75th (8.5** μ**g/m**^3^**)**	**95th (12.0** μ**g/m**^3^**)**
Overall	5th	0.97 (0.95, 1.000)	0.96 (0.93, 1.000)	0.95 (0.90, 0.99)	0.933 (0.88, 0.99)
	95th	0.99 (0.97, 1.002)	0.98 (0.95, 1.003)	0.970 (0.94, 1.004)	0.96 (0.92, 1.005)
Extremely	5th	0.91 (0.82, 1.011)	0.86 (0.73, 1.017)	0.82 (0.66, 1.022)	0.78 (0.59, 1.028)
	95th	0.96 (0.88, 1.039)	0.93 (0.82, 1.063)	0.91 (0.77, 1.08)	0.89 (0.71, 1.107)
Very	5th	0.96 (0.89, 1.033)	0.93 (0.83, 1.053)	0.91 (0.78, 1.070)	0.89 (0.73, 1.09)
	95th	0.98 (0.90, 1.069)	0.97 (0.85, 1.111)	0.96 (0.81, 1.147)	0.95 (0.76, 1.193)
Moderate to late	5th	0.98 (0.96, 1.004)	0.97 (0.93, 1.007)	0.96 (0.91, 1.009)	0.95 (0.887, 1.012)
	95th	1.000 (0.98, 1.018)	1.001 (0.97, 1.029)	1.001 (0.97, 1.038)	1.001 (0.96, 1.049)

The relationship between particulate matter ≤ 2.5 microns (PM_2.5_) and sPTB was modeled as linear and for mean temperature (T_avg_) as natural cubic spline with three degrees for freedom and median temperature (18.1 C°) as the reference point. The interaction was assessed by introducing a product term between these two variables in the model. The effect of temperature on PM_2.5_ is shown at low (5th percentile = 11.5°C) and high (95th percentile = 24.5°C) temperature levels. Hazard ratios are compared to the lowest value of PM_2.5_ = 0.82 μg/m^3^ with temperature at its median level (18.1°C). All models were adjusted for year and month of conception, weekdays vs. weekends/holidays, maternal age, parity, smoking during pregnancy, area-level socio-economic status of the mother, and exposure to longer term (i.e., exposure from conception until the week before delivery) gestational mean temperature T_avg_ and PM_2.5_.

There was no clear pattern visible for the interaction effects of short-term exposure to PM_2.5_ on the association between short-term exposure to temperature and sPTB. For overall and extremely sPTB, HRs increased for cold and heat as PM_2.5_ increased from low (3.7 μg/m^3^) to high (12.0 μg/m^3^)values (5th vs. 95th percentile) (Table 4; Supplementary Figure S7). For very sPTB, the point estimates for T_avg_ showed a decreased risk at the 5th percentile level of PM_2.5_ and an increased risk for PM_2.5_ at the 95th percentile [from 0.75 (95% CI: 0.59, 0.97) to 1.05 (95% CI: 0.80, 1.35)]. Hazard ratios for moderate-to-late sPTB increased marginally with increasing PM_2.5_ values for hot temperatures. For all sPTB groups other than very sPTB at low temperatures and overall sPTB at high temperatures, confidence intervals for the joint effects overlapped and the confidence intervals contained the point estimate from the comparison group.

**Table 4:** Spontaneous preterm birth (sPTB) hazard ratios for the interaction effect between mean T_avg_ and mean PM_2.5_ in the week before delivery for all live births in the Greater Metropolitan Region of New South Wales between 1 January 2001 and 31 December 2019.

		**Temperature percentiles**			
**sPTB group**	**PM**_2.5_ **percentiles**	**5th (11.5**°**C)**	**25th (14.1**°**C)**	**75th (21.6**°**C)**	**95th (24.5**°**C)**
Overall	5th	0.92 (0.86, 0.98)	0.94 (0.90, 0.98)	1.085 (1.05, 1.121)	1.180 (1.108, 1.257)
	95th	0.97 (0.90, 1.034)	0.92 (0.88, 0.97)	1.143 (1.100, 1.189)	1.263 (1.189, 1.343)
Extremely	5th	1.033 (0.77, 1.394)	0.95 (0.78, 1.158)	1.101 (0.95, 1.274)	1.134 (0.86, 1.502)
	95th	1.080 (0.771, 1.511)	0.99 (0.81, 1.220)	1.154 (0.96, 1.39)	1.409 (1.066, 1.862)
Very	5th	0.75 (0.59, 0.97)	0.88 (0.75, 1.037)	1.113 (0.98, 1.261)	1.322 (1.042, 1.677)
	95th	1.052 (0.82, 1.35)	0.93 (0.79, 1.099)	1.176 (1.015, 1.362)	1.299 (1.032, 1.636)
Moderate to late	5th	1.000 (0.929, 1.07)	0.99 (0.95, 1.041)	1.013 (0.98, 1.05)	1.029 (0.962, 1.100)
	95th	0.98 (0.91, 1.054)	0.98 (0.94, 1.027)	1.034 (1.06, 1.078)	1.072 (1.004, 1.144)

The relationship between particulate matter ≤ 2.5 microns (PM_2.5_) and sPTB was modeled as linear and for mean temperature (T_avg_) as natural cubic spline with three degrees for freedom and median temperature (18.1°C) as the reference point. The interaction was assessed by introducing a product term between these two variables. All hazard ratios are relative to PM_2.5_ at its median level (6.83 μg/m^3^) and T_avg_ at its median level (18.1°C). All models were adjusted for year and month of conception, weekdays vs. weekends/holidays, maternal age, parity, smoking during pregnancy, area-level socio-economic status of the mother, exposure to short-term (the week before delivery), longer term gestational (exposure across the entire pregnancy except for the last week before delivery) T_avg_, and longer term gestational PM_2.5_.

### Sensitivity analyses

The decreased risk estimates between short-term exposure to PM_2.5_ and all sPTB groups remained consistent throughout almost all models tested in the sensitivity analysis (Supplementary Tables S5, S9). The one exception was for very sPTB with PM_2.5_ modeled as non-linear. In this model, HRs increased per unit increment in PM_2.5_ (μg/m^3^) until the 25th percentile, and thereafter, HRs started decreasing (Supplementary Table S9).

Similar J- and U-shaped relationships between temperature and different sPTB groups as in the main analysis reappeared in the sensitivity tests (Supplementary Tables S6, S10).

The interaction effect of temperature on PM_2.5_ was similar across most sPTB models in the main and sensitivity analysis—a slight increase in sPTB HRs at high-temperature values was observable compared with lower temperatures (Supplementary Tables S7, S11). In the 4-week exposure model though, for very sPTB, the HRs decreased as temperature increased.

Finally, concerning the effects of PM_2.5_ on temperature, all sensitivity models indicated increased HRs for the cold effects on sPTB when PM_2.5_ changed from the 5th percentile to the 95^th^ percentile (Supplementary Tables S8, S12). The same was usually the case for the effects of increasing PM_2.5_ exposure on heat. The only exceptions were the 4-week exposure model for extremely and very sPTB—the heat HR decreased in this subtype with increasing short-term PM_2.5_ exposure.

## Discussion

### Key results

This population-based retrospective birth cohort study assessed the independent and combined short-term effects of ambient air pollution and temperature on sPTB while controlling for longer term gestational exposure.

Our study found that the risk of sPTB did not increase with increasing short-term PM_2.5_ exposure. However, in this low ambient air pollution environment, longer term gestational exposure to PM_2.5_ was associated with a small increase in the risk of sPTB. The risk of sPTB was also found to increase following acute exposure to low and high ambient temperatures.

Short-term cold and heat effects of temperature on sPTB were higher in the presence of higher acute PM_2.5_ pollution levels.

Investigation of effects by different subtypes of sPTB found that the risk of earlier (extremely and very preterm) sPTBs was more strongly associated with increased acute heat exposure. Furthermore, extremely sPTB was also most susceptible to acute cold effects.

### Strengths

To the best of our knowledge, this is the first study to estimate the short-term joint effects of cold and heat with air pollution on sPTB. Other studies have either assessed only the effects of short-term exposure to heat waves and air pollution (21, 22) or the longer term interaction effect (20). We used a large and high-quality birth cohort dataset, including 19 years of data from the most populous region in Australia. As this study specifically aimed to assess whether short-term environmental exposures could trigger labor, we only considered sPTB as the main outcome in our study. This is important because the underlying mechanisms for which environmental exposures affect pregnancies might be different for sPTB and medically initiated PTB (13). Few other studies have been able to censor (or exclude) non-spontaneous PTB in their analysis. By applying a Cox proportional hazards model, as opposed to other common methods such as time series or case-crossover studies, we accounted for the increased likelihood of giving birth as pregnancy progresses. We considered all three PTB subtypes in our analysis, which many other studies have not been able to do due to their lack of statistical power. Compared to many other Australian studies on PTB [e.g., (36, 59–61)], we had a more spatially resolved outdoor exposure assessment for temperature because we did not rely solely on weather station data; rather, we used a gridded dataset to assign more precise exposures in each geographical location at an SA2 level.

### Limitations

We controlled for area-level socio-economic status, rather than individual-level socio-economic status, which may result in residual confounding. Furthermore, as we did not consider stillbirths, we might have introduced live birth bias into our study. This is a selection bias that can occur if the exposure of interest influences the chances for live births, and if at the same time, there are some other common causes for preterm births and stillbirths which were not considered in a study (39). Another way of how we might have introduced live birth bias is through the depletion of susceptible pregnancies, which occurs if the exposure of interest results in an early pregnancy loss amongst pregnancies that would have otherwise resulted in preterm births (39). However, any such bias would have biased our associations toward null because it preferentially depletes pregnancies exposed to the risk factor studied.

### Interpretation

Although biologically plausible, research has not yet explicitly studied the biological pathway of independence (62–64) and joint effects of temperature and air pollution on PTB. Particulate matter and low and high temperatures may have synergistic effects because they act on common pathophysiological pathways (65). Heat, cold, and PM exposure have been associated with oxidative stress, systemic inflammation, and elevated blood viscosity, which all, in turn, cascade into a chain of processes in the body, which can trigger labor (16, 49, 63). Furthermore, the thermoregulatory system responds to heat stress by increasing sweating, minute ventilation, and cardiac output, all of which tend to increase the uptake and distribution of air pollutants in the body and alter the physiological response to toxic agents possibly increasing predisposition to air pollution (65–67). Cold and air pollution has been linked to maternal hypertensive diseases during pregnancy an important cause of PTB (2, 68–70). Furthermore, cold reduces respiratory mucociliary function and consequently obstructs the clearance of fine particles (71, 72).

Our results broadly concur with similar studies from a range of locations worldwide on the risk of PTB and short-term exposure to cold or hot ambient temperatures. It is noteworthy that some of the studies investigating both cold and heat exposures only found an adverse risk association with heat. For instance, a study from the Southern District of Israel with a semi-arid climate that assessed exposure to low and high temperatures by gestational weeks in a Cox proportional hazard analysis found that late PTB (after week 31) was associated with the 5th temperature quintile relative to the middle quintile [HR = 1.31 (95% CI: 1.11, 1.56)]. There was no association with lower temperature quintiles or earlier PTB (73). A recent study from 2022, which assessed temperature effects in NSW, Australia, found an increased association between overall sPTB and only high temperatures. In their time-series analysis, the cumulative effect of the relative risk of sPTB for mean daily temperature over a lag of 7 days was 1.16 (95% CI: 1.08, 1.20) at the 95*t*h percentile (25°C) compared to the median temperature (17°C) (74). This study did not find any cold effects, whereas, in our study, we do find some adverse effects of extreme cold on overall, extremely, and moderate-to-late sPTBs. However, the hazardous effects of heat were more pronounced and clearer. A reason for this could be the temperate climate with warm summers and no extreme winters in the GMR of NSW. In such a context, cold-related health effects can be lower (75, 76). Importantly, it is worth noting that other studies have reported cold effects. For example, a study based in subtropical Guangzhou, China, assessed the association with temperature for several time windows in a survival analysis and found an association with an increase in risk for PTB for cold and heat for 4 weeks before birth (51). Compared to the median temperature, the HR for cold was 1.13 (95% CI: 1.07, 1.9) and for heat 1.08 (95% CI: 1.02, 1.3) in that study.

Our study suggests that the risk for sPTB did not increase with increasing short-term exposure to PM_2.5_. This is consistent with the negative and null findings of other studies in similar populations. One study from Sydney, Australia, which assessed the relationship between air pollution exposure 1–3 months before birth found a negative association between PM_2.5_ and overall PTB (52). The odds ratio for the 1-month relationship was 0.98 (95%. CI: 0.96–1.00). A study from Brisbane, Australia, assessed the short-term effect (up to 3 days) before birth. Similarly, to the previous study, it found a statistically non-significant reduced cumulative odds ratio (effect estimates not reported) for PM_2.5_ exposure and overall PTB (47). A negative relationship with PM_2.5_ exposure up to a lag of 6 days was also reported in studies outside of Australia. A study from Canada in 24 cities reported in their Cox proportional hazard analysis that per 7.4 μg/m^3^ increase in PM_2.5_ at lag 6 an HR of 0.998 (95% CI: 0.987, 1.008) (77). It is noteworthy that this and the prior two Australian studies were conducted in relatively low air pollution environments, and they did not control for longer term gestational exposure to PM_2.5_ as in our study. Two other studies from Guangzhou, China, where ambient air pollution is higher [e.g., from 1 September 2006 to 11 July 2013: 70.4 μg/m^3^ (SD: 60.6 μg/m^3^); 52], included mutual adjustments for short-term and longer term PM_2.5_ exposure (22, 54) and both of these studies found a positive, but not significant, association between short-term PM_2.5_ and PTB. For example, one of these two studies applied a time-series analysis and found for the longer term effect a 3.16% (95% CI: 1.95%, 4.39%) increase per 10-μg/m^3^ increment in PM_2.5_. While for the acute relationship, a much lower effect with 0.19% (95% CI: −0.26%, 0.65%) per 10-μg/m^3^ increment in PM_2.5_ was found (54).

Systematic reviews show that the evidence on the adverse effects of PM_2.5_ on PTB is the strongest for entire pregnancy exposures (2, 5). Considering our results and the evidence from other studies, it might be that PM_2.5_ exposure does not have any considerable short-term acute effects on sPTB compared with longer term gestational exposure. For sPTB, there might be a more indirect mechanism between PM_2.5_ and the way labor is triggered, requiring cumulative exposure to PM_2.5_, rather than acute exposure, whereas temperature may contribute in an acute manner.

Only three other studies have assessed the joint effects of air pollution and temperature on PTB. Two of these studies assessed the synergistic effects between heat waves and air pollution in California, USA, and Guangzhou, China, by assessing relative excess risk due to interaction (RERI) (21, 22). The Guangzhou study did not find any significant association for the joint effect; however, there was an indication that effects for less intense heat waves in combination with PM_2.5_ exposure were larger than expected [i.e., synergistic; positive additive interactions (RERI > 0)] and the joint effects with more extreme heat waves had negative additive interaction (RERIs < 0), possibly due to heat-mitigating behaviors occurring during extreme heat. The Californian study on the other hand found a positive additive interaction (RERI > 0) between some of their heat wave indicators and PM_2.5_ exposure (21). The third study from Brisbane, Australia, found that the hazardous effect of PM_2.5_ was greater at moderate and low temperatures than at high temperatures, potentially indicating some heat mitigating behaviors on hot days like in the Guangzhou study (20). We found some notable patterns for a two-way interaction between short-term exposure to temperature and air pollution; however, the moderating effects were mostly rather weak and not always persistent. The confidence intervals were often wide or contained the effect estimates of the comparison group.

Our main and sensitivity analysis showed that the cold and heat HRs for extremely and the heat HRs for very sPTB were higher than moderate-to-late sPTB, indicating potential susceptibility to temperature already at earlier stages of the pregnancy. Furthermore, extremely and very sPTBs had larger HRs for longer term gestational PM_2.5_ exposure. These two subtypes were also more reactive to the joint effects of temperature and PM_2.5_. Concerning which preterm subtype is more vulnerable to temperature and air pollution exposure, non-environmental factors, such as infections and lifestyles, rather than environmental factors, may play an increasingly important role in earlier preterm births, such as extremely and very PTB (78, 79). However, for later PTB, such as moderate-to-late PTB environmental stressors are more likely to contribute (78, 79). Whereas, some studies confirm this hypothesis for the heat effects (22, 73), others find like in our study that earlier PTB is more susceptible [e.g., (51)]. Further studies are needed to elucidate the most vulnerable subtypes, as decreasing gestational age is associated with increasing mortality and health complications (8).

Evidence on which exposure window during pregnancy is susceptible to temperature and air pollution exposure is mixed (2, 4–6, 15–17). In this study, we chose to focus on ambient PM_2.5_ and ambient temperature in the last gestational week before birth due to the existing evidence of heat on PTB during this period. The susceptible time window for each sPTB subtype from each individual environmental stressor—cold, heat, and air pollution—may, however, differ, and this needs to be considered by future studies, including their lagged and cumulative effects, and the nature of the joint effects.

## Conclusion

The literature does not provide consistent guidance on which window during pregnancy is most vulnerable to temperature and PM_2.5_ exposure. This study adds to the evidence that short-term exposure to ambient cold and heat and longer term gestational exposure to ambient PM_2.5_ is associated with an elevated risk of sPTB. The short-term hazardous effects were more pronounced and evident for heat than for cold. Like other studies in a low air pollution setting, we did not find any evidence of the hazardous effects of short-term exposure to PM_2.5_.

Different preterm subtypes are differently susceptible to the independent and joint effects of exposure to ambient air pollution and temperature. Earlier sPTB subtypes seemed to be the most sensitive to the environmental risk exposures assessed in this study.

## Data availability statement

The data analyzed in this study is subject to the following licenses/restrictions: the data needs to be requested specifically from the New South Wales Ministry of Health. The authors of this manuscript are not allowed to share the data with a third party. Requests to access these datasets should be directed to the New South Wales Ministry of Health.

## Ethics statement

The studies involving humans were approved by University of New South Wales Human Research Low Risk Ethics Advisory Committee Panel E (Reference Number: HC200817). The studies were conducted in accordance with the local legislation and institutional requirements. Written informed consent for participation was not required from the participants or the participants' legal guardians/next of kin in accordance with the national legislation and institutional requirements.

## Author contributions

TS contributed to conceptualization, methodology, software, writing—original draft preparation, and formal analysis. BJ contributed to resources and writing—review and editing. SH was involved in methodology and writing—review and editing. GM contributed to writing—review and editing. KM and DG were involved in supervision and writing—review and editing. JK contributed to supervision and methodology. EJ contributed to supervision, resources, methodology, and writing—review and editing. All authors contributed to the article and approved the submitted version.
