# Supplementary material for: Acute air pollution and temperature exposure as independent and joint triggers of spontaneous preterm birth in New South Wales, Australia: a time-to-event analysis
Source: Front Public Health. 2023 Nov 30;11:1220797. doi: 10.3389/fpubh.2023.1220797 (PMC10720724; doi:10.3389/fpubh.2023.1220797)
Supplement: Supplementary file 1 [file Data_Sheet_1.pdf]

Acute Air Pollution and Temperature Exposure as Independent and Joint Triggers of Spontaneous Preterm Birth in New South Wales, Australia: A Time-to-Event Analysis

Authors: Tanya Singh[1,2*], Bin Jalaludin[3,4], Shakoor Hajat[5], Geoffrey G Morgan[6,7,8,9], Katrin Meissner[1,2], John Kaldor[10], Donna Green[1,2], Edward Jegasothy[6,7]

[1] Climate Change Research Centre, University of New South Wales, Sydney, Australia

[2] Australian Research Council Centre of Excellence for Climate Extremes, University of New South Wales, Sydney, Australia

[3] School of Population Health, University of New South Wales, Kensington, NSW, 1871, Australia

[4] Ingham Institute for Applied Medical Research, University of New South Wales, Sydney, Australia

[5] Centre on Climate Change & Planetary Health, London School of Hygiene & Tropical Medicine, London, United Kingdom

[6] School of Public Health, Faculty of Medicine and Health, University of Sydney, Camperdown, NSW 2006, Australia

[7] University Centre for Rural Health, Faculty of Medicine and Health, University of Sydney, Lismore, NSW 2480, Australia

[8] Healthy Environments and Lives (HEAL) National Research Network, Australian National University, Canberra, Australian Capital Territory, Australia

[9] Centre for Safe Air, National Health and Medical Research Council Centre of Excellence, University of Tasmania, Hobart, Tasmania, Australia

[10] Kirby Institute, University of New South Wales, Sydney, Australia

[*] Corresponding author. Climate Change Research Centre, University of New South Wales, Sydney, Australia, Level 4, Mathews Building, The University of New South Wales Sydney NSW 2052, email: tanya.singh@unsw.edu.au

Figure S1: Australia map showing New South Wales (NSW), Greater Metropolitan Region (GMR) and subregions and major cities within the subregions. Map inset shows position of NSW (grey) within Australia and position of the GMR (red) within NSW.

Figure S2: Flowchart of inclusion and exclusion criteria for study population (mothers who gave a live birth within the Greater Metropolitan Region of New South Wales, Australia, between 1 January 2001 and 31 December 2019)

Figure S3: Directed Acyclic Graph (DAG) regarding the association between acute ambient air temperature and acute $PM_{2.5}$ (particulate matter less than or equal to 2.5 microns in aerodynamic diameter) and preterm birth.

Table S1: Descriptive statistics for exposure to $PM_{2.5}$ (μg/m^3) during the last gestational week before birth by season for all mothers who gave a live birth within the Greater Metropolitan Region of New South Wales, Australia, between 1 January 2001 and 31 December 2019

Figure S4: Spatial distribution of mean $PM_{2.5}$ (μg/m^3) the week before birth by statistical area level 2 (SA2) for all mothers (N= 1,318,570) who gave a live birth within the Greater Metropolitan Region of New South Wales, Australia, between 1 January 2001 and 31 December 2019

Figure S5: Spatial distribution of mean temperature *(°C)* the week before birth by statistical area level 2 (SA2) for all mothers (N= 1,318,570) who gave a live birth within the Greater Metropolitan Region of New South Wales, Australia, between 1 January 2001 and 31 December 2019

Table S2: Pearson correlation matrix for different exposure types for all live births (N= 1,318,570) in the Greater Metropolitan Region of New South Wales between 1 January 2001 and 31 December 2019

Table S3: Hazard Ratios for the short-term (last gestational week before delivery) and longer-term gestational exposure (exposure across the entire pregnancy except for the last week before delivery) to $PM_{2.5}$ for all spontaneous preterm birth (sPTB) groups in the Greater Metropolitan Region of New South Wales between 1 January 2001 and 31 December 2019

Table S4: Hazard Ratios for the short-term (in the last gestational week before delivery) exposure to mean temperature (°C) for all spontaneous preterm birth groups in the Greater Metropolitan Region of New South Wales between 1 January 2001 and 31 December 2019

Figure S6: Spontaneous preterm birth (sPTB) hazard ratios (HR) for the interaction between mean $PM_{2.5}$ and mean T_{avg} in the week before delivery derived from the Cox proportional hazard model.

Figure S7: Spontaneous preterm birth (sPTB) hazard ratios (HR) for the interaction between mean $PM_{2.5}$ and mean T_{avg} in the week before delivery derived from the Cox proportional hazard model

Table S5: Hazard ratios and confidence intervals for the short-term exposure to 4-week mean $PM_{2.5}$ (in the last four weeks before delivery) in the 4-week exposure model for all spontaneous preterm birth (sPTB) groups in the Greater Metropolitan Region of New South Wales between 1 January 2001 and 31 December 2019

Table S6: Hazard ratios and confidence intervals for the short-term exposure to 4-week mean temperature (T_{avg}) (in the last four weeks before delivery) in the 4-week exposure model for all spontaneous preterm birth (sPTB) groups in the Greater Metropolitan Region of New South Wales between 1 January 2001 and 31 December 2019.

Table S7: Spontaneous Preterm (sPTB) birth hazard ratios and 95% confidence intervals for the interaction effect of 4-week T_{avg} on 4-week $PM_{2.5}$ in the last four gestational weeks before delivery for all live births in the Greater Metropolitan Region of New South Wales between 1 January 2001 and 31 December 2019.

Table S8: Spontaneous Preterm (sPTB) birth hazard ratios and 95% confidence intervals for the interaction effect of 4-week $PM_{2.5}$ on 4-week T_{avg} in the last four gestational weeks before delivery for all live births in the Greater Metropolitan Region of New South Wales between 1 January 2001 and 31 December 2019.

Table S9: Hazard ratios and confidence intervals for short-term exposure to $PM_{2.5}$ as a natural cubic spline term for all spontaneous preterm birth (sPTB) groups in the Greater Metropolitan Region of New South Wales between 1 January 2001 and 31 December 2019 for $PM_{2.5.}$

Table S10: Hazard ratios for the short-term (in the last gestational week before delivery) exposure to T_{avg} with exposure to $PM_{2.5}$ modelled as a non-linear term for all spontaneous preterm birth (sPTB) groups in the Greater Metropolitan Region of New South Wales between 1 January 2001 and 31 December 2019.

Table S11: Spontaneous preterm birth (sPTB) hazard ratios and 95% confidence intervals for the interaction effect between mean T_{avg} and mean $PM_{2.5}$, with $PM_{2.5}$ as a non-linear term in the week before

delivery for all live births in the Greater Metropolitan Region of New South Wales between 1 January 2001 and 31 December 2019.

Table S12: Spontaneous Preterm (sPTB) birth hazard ratios and 95% confidence intervals for the interaction effect of $PM_{2.5.}$ as non-linear term on T_{avg} for all live births in the Greater Metropolitan Region of New South Wales between 1 January 2001 and 31 December 2019.

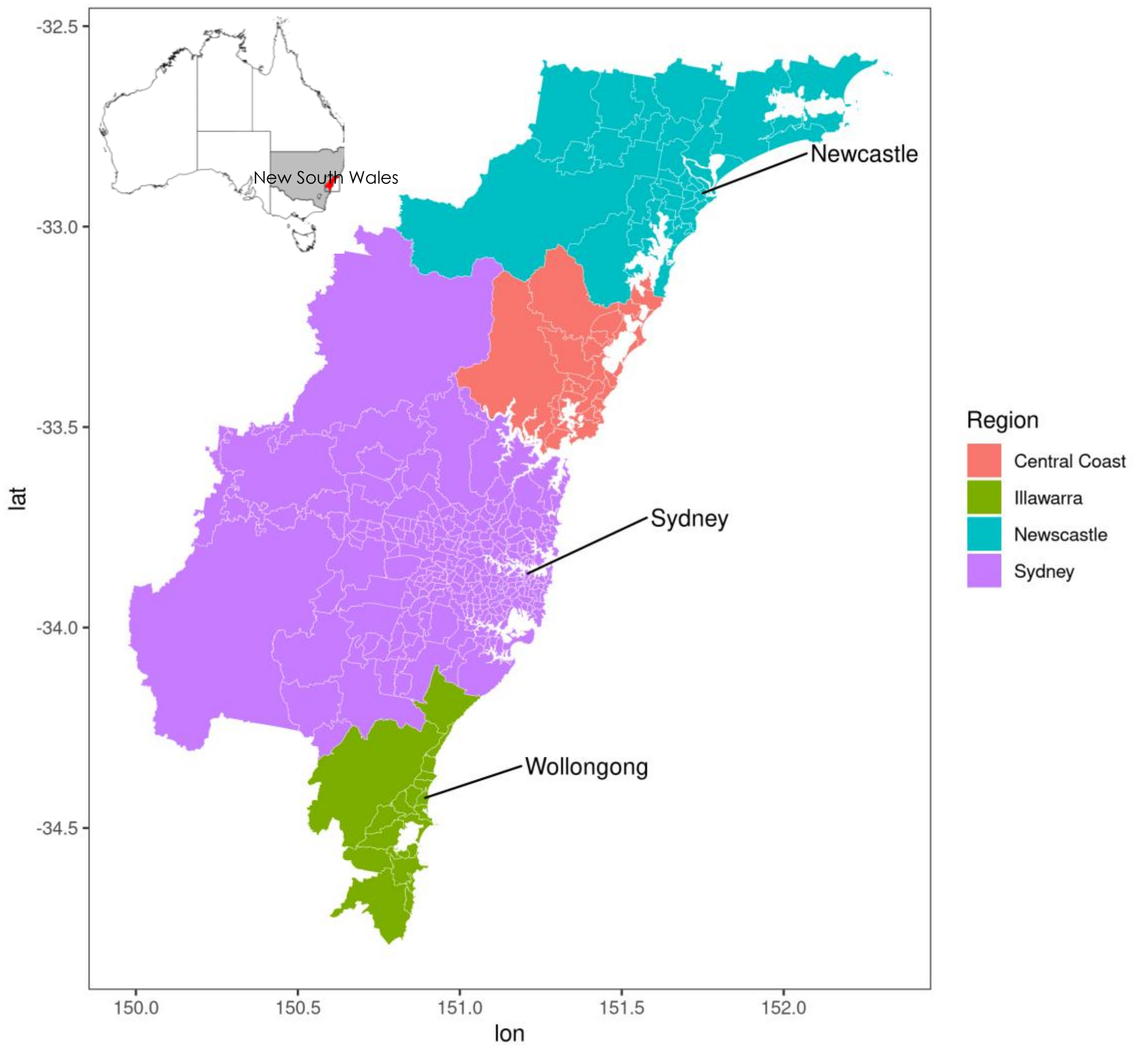

Figure S1: Australia map showing New South Wales (NSW), Greater Metropolitan Region (GMR) and subregions and major cities within the subregions. The white lines show the boundaries of Statistical Area Level 2. The map inset shows the position of NSW (grey) within Australia and the position of the GMR (red) within NSW.

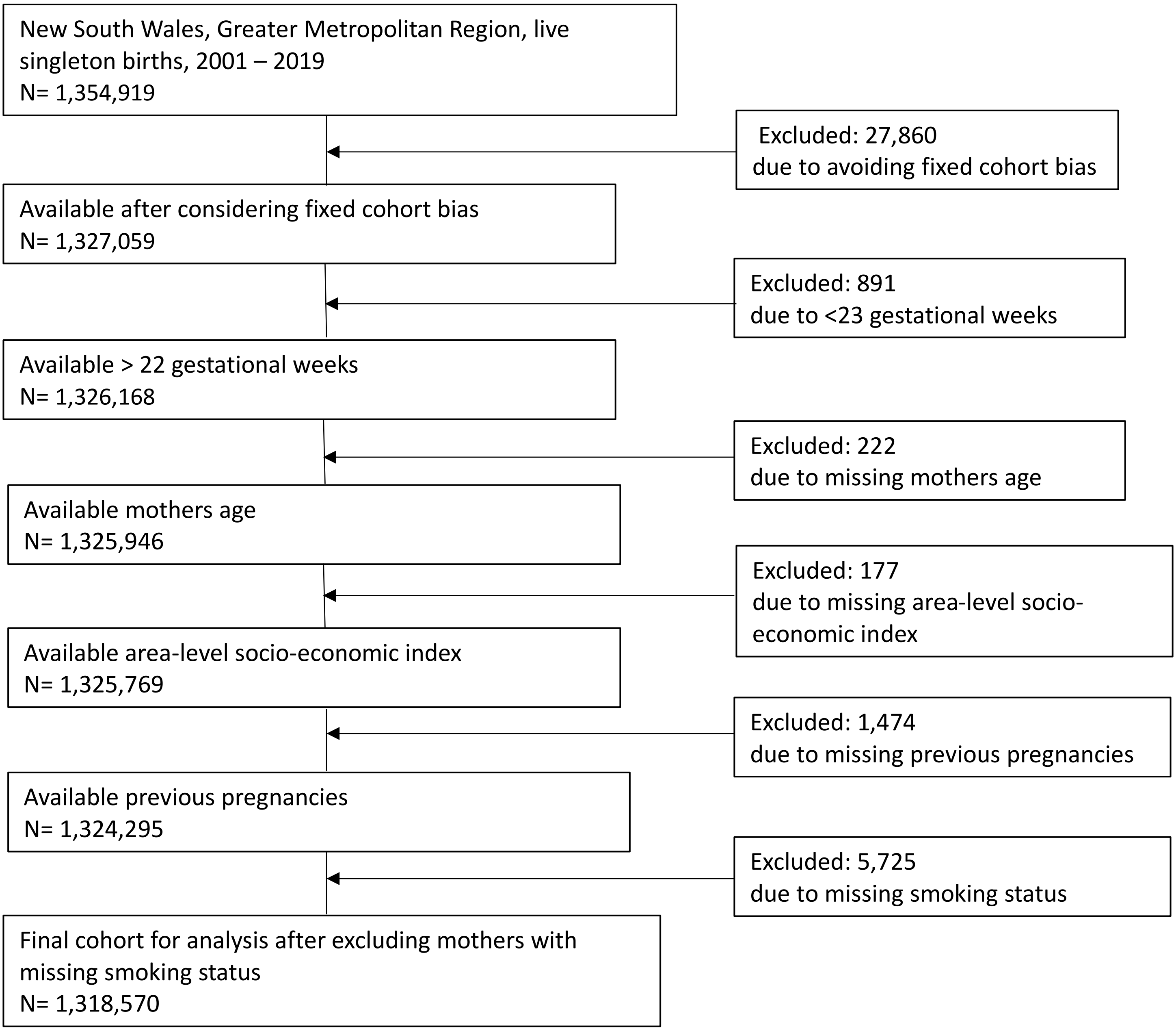

Figure S2: Flowchart of inclusion and exclusion criteria for study population (mothers who gave a live birth within the Greater Metropolitan Region of New South Wales, Australia, between 1 January 2001 and 31 December 2019)

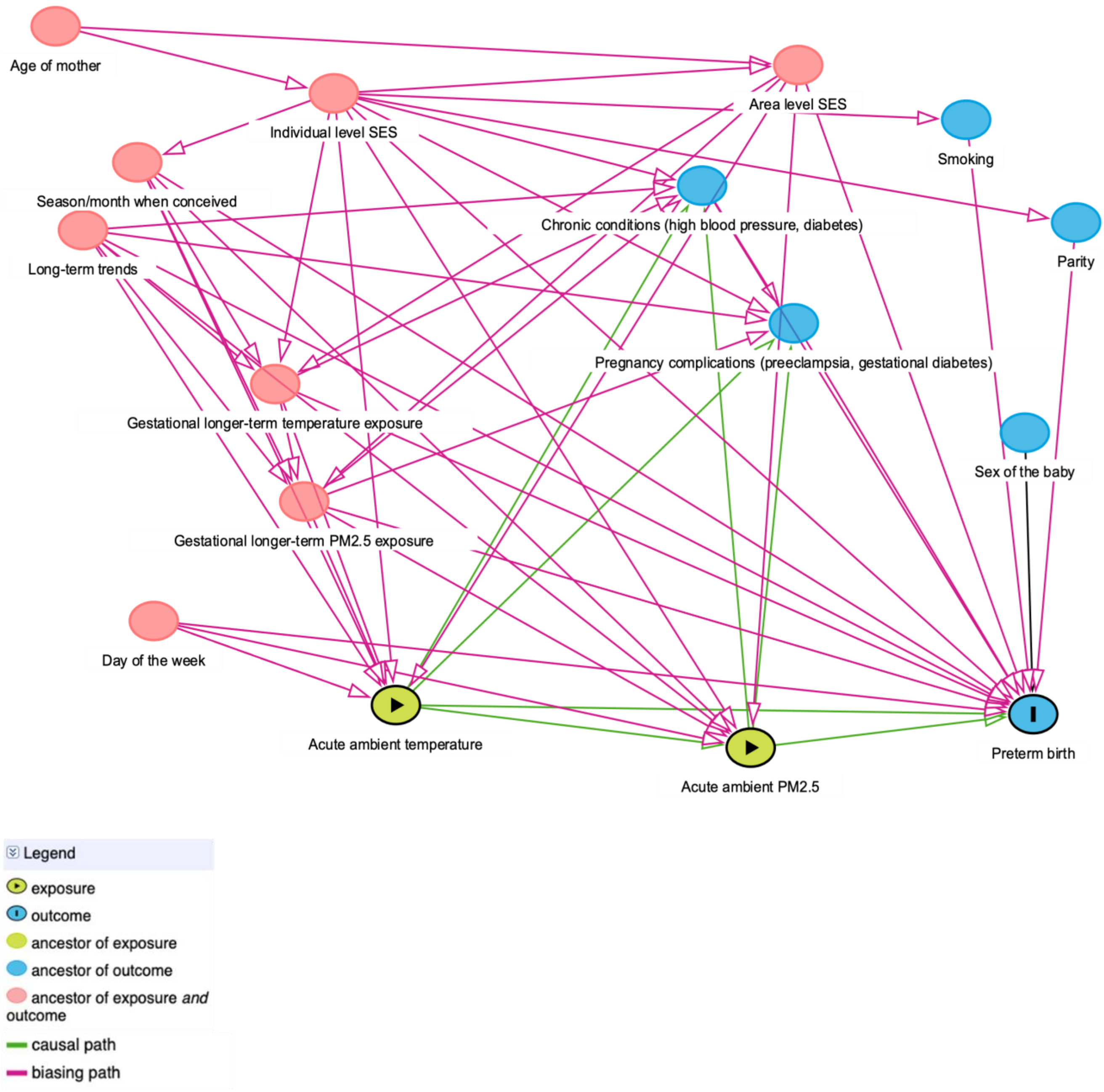

Figure S3: Directed Acyclic Graph (DAG) regarding the association between acute ambient air temperature and acute $PM_{2.5}$ (particulate matter less than or equal to 2.5 microns in aerodynamic diameter) and preterm birth.

Table S1: Descriptive statistics for exposure to $PM_{2.5}$ (μg/m^3) during the last gestational week before birth by season for all mothers who gave a live birth within the Greater Metropolitan Region of New South Wales, Australia, between 1 January 2001 and 31 December 2019

Birth type	Birth season	Mean	SD	Minimum	25th	Median	75th	Maximum
All births (n= 1,318,570)	Spring	7.32	3.45	1.81	5.49	6.78	8.38	63.51
	Summer	7.58	4.42	1.81	5.60	6.97	8.51	83.95
	Autumn	6.80	2.39	0.82	5.20	6.48	7.91	29.40
	Winter	7.62	2.96	1.25	5.50	7.22	9.24	29.40
Overall sPTB (n= 38,900)	Spring	7.26	3.49	2.11	5.36	6.65	8.30	63.51
	Summer	7.49	4.43	1.98	5.56	6.87	8.33	83.95
	Autumn	7.15	2.68	0.83	5.37	6.73	8.39	28.09
	Winter	7.26	2.75	1.64	5.24	6.96	8.85	26.65
Extremely sPTB (n= 2,001)	Spring	7.01	2.90	2.28	5.14	6.35	8.23	28.21
	Summer	7.36	3.24	1.98	5.63	6.94	8.39	40.67
	Autumn	7.04	2.51	2.26	5.41	6.76	8.32	19.87
	Winter	7.04	2.57	1.84	5.17	6.76	8.57	19.63
Very sPTB (n= 3,059)	Spring	7.42	3.76	2.36	5.42	6.66	8.36	48.02
	Summer	7.28	3.79	2.87	5.50	6.85	8.30	62.54
	Autumn	6.99	2.43	0.83	5.40	6.59	8.14	19.44
	Winter	7.29	2.69	1.98	5.32	7.07	9.03	16.87
Moderate-to-late sPTB (n= 33,840)	Spring	7.26	3.50	2.11	5.37	6.66	8.29	63.51
	Summer	7.51	4.55	2.11	5.56	6.87	8.33	83.95
	Autumn	7.17	2.72	1.02	5.37	6.74	8.42	28.09
	Winter	7.27	2.76	1.64	5.24	6.96	8.86	26.65

Note: sPTB, spontaneous preterm birth; SD, standard deviation; n, number. All births include live spontaneous preterm births, all live non-spontaneous births and live term births (i.e. gestational age > 36 weeks). It should be noted that this table does not represent the comparisons made in each of the Cox proportional hazard models. Term and non-spontaneous births together, however, represent most of all births in our dataset and therefore are a good approximation for the comparisons used in each model.

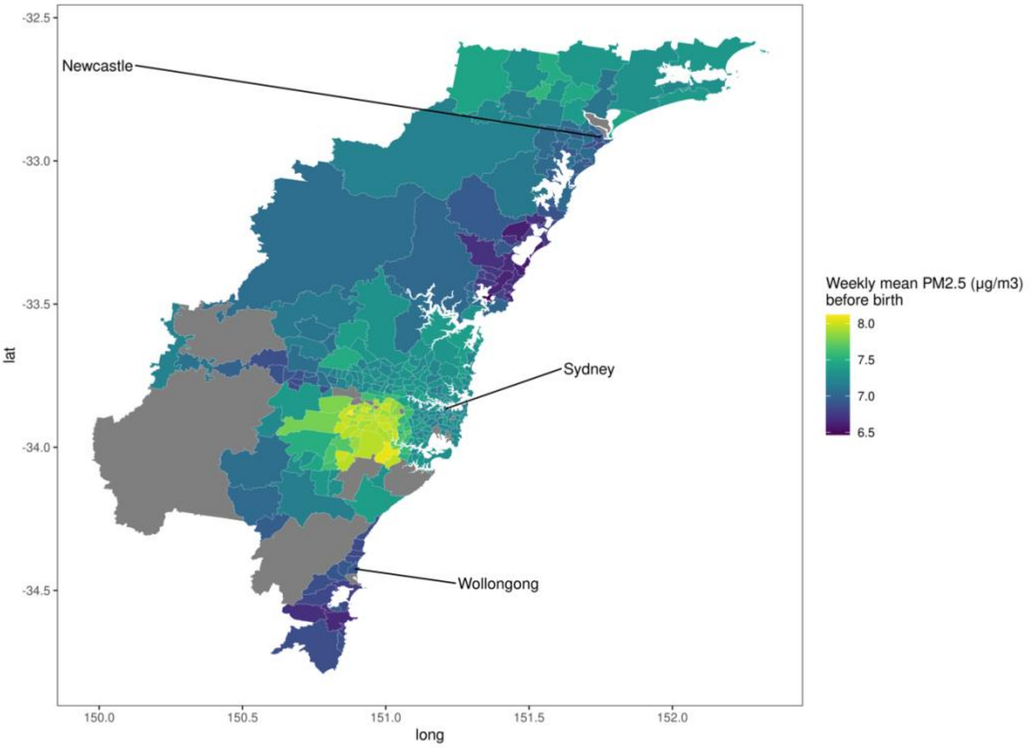

Figure S4: Spatial distribution of mean $PM_{2.5}$ ($\mu g/m^3$) the week before birth by statistical area level 2 (SA2) for all mothers (N= 1,318,570) who gave a live birth within the Greater Metropolitan Region of New South Wales, Australia, between 1 January 2001 and 31 December 2019

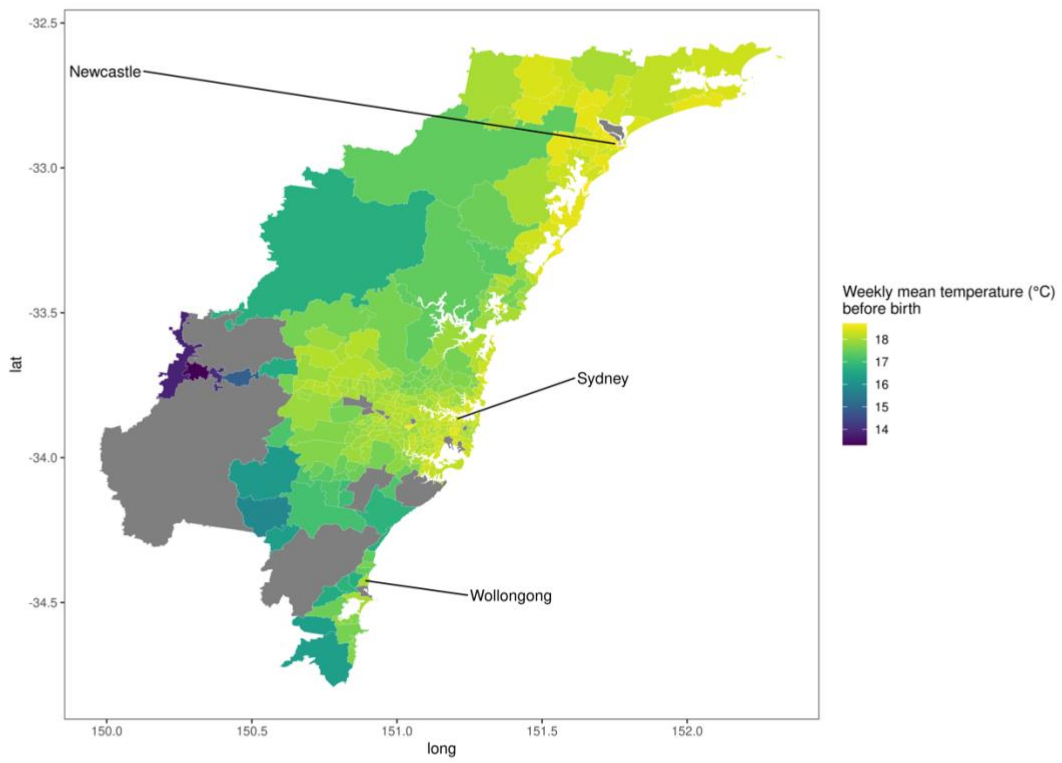

Figure S5: Spatial distribution of mean temperature *(°C)* the week before birth by statistical area level 2 (SA2) for all mothers (N= 1,318,570) who gave a live birth within the Greater Metropolitan Region of New South Wales, Australia, between 1 January 2001 and 31 December 2019

Table S2: Pearson correlation matrix for different exposure types for all live births (N= 1,318,570) in the Greater Metropolitan Region of New South Wales between 1 January 2001 and 31 December 2019

		**Last gestational week before birth**		**Last 4 gestational weeks before birth**		**Longer-term gestational exposure**			
	**Exposure variable**	**$PM_{2.5}$**	**T_{avg}**	**$PM_{2.5}$**	**T_{avg}**	**$PM_{2.5}$**	**T_{avg}**	**4-week $PM_{2.5}$**	**4-week T_{avg}**
**Last gestational week before birth**	**$PM_{2.5}$**	1.00	0.08	0.62	0.01	0.26	0.02	0.23	0.02
	**T_{avg}**	0.08	1.00	0.01	0.95	0.06	-0.43	0.07	-0.58
**Last 4 gestational weeks before birth**	**$PM_{2.5}$**	0.62	0.01	1.00	0.02	0.49	0.03	0.37	0.03
	**T_{avg}**	0.01	0.95	0.02	1.00	0.07	-0.30	0.07	-0.47
**Longer-term gestational exposure**	**$PM_{2.5}$**	0.26	0.06	0.49	0.07	1.00	0.07	0.99	0.05
	**T_{avg}**	0.02	-0.43	0.03	-0.30	0.07	1.00	0.07	0.98
	**4-week $PM_{2.5}$**	0.23	0.07	0.37	0.07	0.99	0.07	1.00	0.05
	**4-week T_{avg}**	0.02	-0.58	0.03	-0.47	0.05	0.98	0.05	1.00

Note: p-values for all correlations <.001

$PM_{2.5}$ = particulate matter less than or equal to 2.5 microns in aerodynamic diameter; T_{avg} = mean temperature; longer-term gestational exposure = entire pregnancy excluding the week before delivery for $PM_{2.5}$ and T_{avg} and entire pregnancy excluding the last 4 weeks before birth for 4-week $PM_{2.5}$ and 4-week T_{avg}

Table S3: Hazard Ratios for the short-term (last gestational week before delivery) and longer-term gestational exposure (exposure across the entire pregnancy except for the last week before delivery) to $PM_{2.5}$ for all spontaneous preterm birth (sPTB) groups in the Greater Metropolitan Region of New South Wales between 1 January 2001 and 31 December 2019

**Exposure type**	**sPTB group**	**HR $PM_{2.5}$ per µg/m^3**	**HR $PM_{2.5}$ per 5 µg/m^3**	**HR $PM_{2.5}$ per 10 µg/m^3**	**HR $PM_{2.5}$ per IQR [µg/m^3]**
**Week before birth**	Overall (N =38,900)	0.990 (0.987,0.994)	0.952 (0.936,0.968)	0.906 (0.876,0.937)	0.970 (0.960,0.980)
	Extremely (N =2,001)	0.971 (0.954,0.988)	0.862 (0.79,0.941)	0.743 (0.624,0.885)	0.913 (0.865,0.963)
	Very (N =3,059)	0.984 (0.972,0.997)	0.924 (0.867,0.985)	0.854 (0.752,0.971)	0.953 (0.916,0.991)
	Moderate-to-late (N =33,840)	0.997 (0.993,1.000)	0.980 (0.970, 1.000)	0.969 (0.936,1.003)	0.990 (0.980,1.001)
**Longer-term gestational**	Overall	1.013 (1.003,1.024)	1.068 (1.015,1.124)	1.141 (1.03,1.263)	1.023 (1.005,1.041)
	Extremely	1.061 (1.019,1.104)	1.341 (1.099,1.638)	1.799 (1.207,2.681)	1.111 (1.034,1.194)
	Very	1.038 (1.003,1.074)	1.205 (1.017,1.426)	1.451 (1.035,2.035)	1.068 (1.006,1.133)
	Moderate-to-late	1.000 (0.989,1.011)	1.000 (0.948,1.056)	1.001 (0.898,1.115)	1.000 (0.982,1.019)

Note: N, number of spontaneous preterm birth in each model; HR, hazard ratio; IQR, interquartile range. IQR for $PM_{2.5}$ the last week before delivery = 3.08 µg/m^3 and for longer-term gestational $PM_{2.5}$ 1.73 µg/m^3 All Cox proportional hazard models were adjusted for year and month of conception, weekdays vs. weekends /holidays, maternal age, parity, smoking during pregnancy, area level socio-economic status of the mother and exposure to short- and longer-term gestational $PM_{2.5}$ and T_{avg}. Both short-term and long-term gestational $PM_{2.5}$ were entered as a linear term in the model and short-term and long-term gestational T_{avg} were non-linear terms.

Table S4: Hazard Ratios for the short-term (in the last gestational week before delivery) to mean temperature (°C) for all spontaneous preterm birth groups in the Greater Metropolitan Region of New South Wales between 1 January 2001 and 31 December 2019

**Exposure type**	**sPTB group**	**Temperature percentiles (°C)**			
		**5th** (11.5)	**25th** (14.1)	**75th** (22.0)	**95th** (24.5)
**Week before birth**	Overall (N =38,900)	0.949 (0.899,1.001)	0.943 (0.91,0.977)	1.104 (1.071,1.137)	1.217 (1.158,1.278)
	Extremely (N =2,001)	1.078 (0.838,1.387)	0.99 (0.835,1.172)	1.118 (0.986,1.268)	1.272 (1.029,1.573)
	Very (N =3,059)	0.869 (0.710,1.063)	0.909 (0.794,1.041)	1.117 (1.003,1.245)	1.257 (1.053,1.501)
	Moderate-to-late (N =33,840)	0.996 (0.939,1.055)	0.993 (0.956,1.032)	1.022 (0.989,1.055)	1.053 (0.999,1.111)

Note: N, number of spontaneous preterm birth in each model. All Cox proportional hazard models were adjusted for year and month of conception, weekdays vs. weekends /holidays, maternal age, parity, smoking during pregnancy, area level socio-economic status of the mother and exposure to short- and longer-term gestational $PM_{2.5}$ and T_{avg}. Both short-term and long-term gestational $PM_{2.5}$ were entered as a linear term in the model and short-term and long-term gestational T_{avg} were modelled as natural cubic splines with 3 degrees of freedom. All hazard ratio estimates are relative to the median weekly mean temperature (18.1°C).

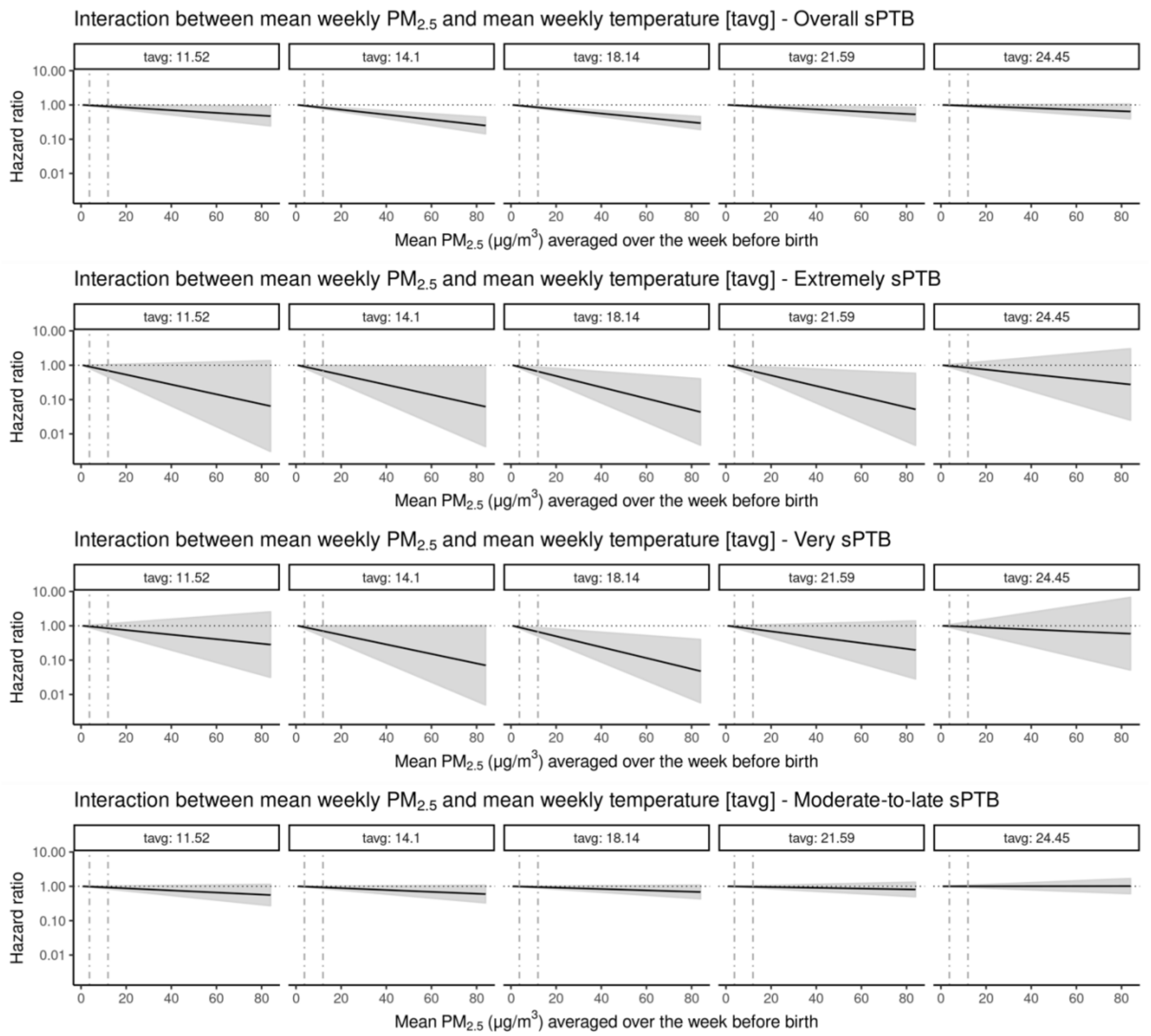

Figure S6: Spontaneous preterm birth (sPTB) hazard ratios (HR) for the interaction between mean $PM_{2.5}$ and mean T_{avg} in the week before delivery derived from the Cox proportional hazard model. The relationship between $PM_{2.5}$ and sPTB was modelled as linear and for T_{avg} as natural cubic spline with three degrees of freedom. The interaction was assessed by introducing a product term between these two variables. The figure shows how the HR change for the association between $PM_{2.5}$ and sPTB as temperature increases from its 5th percentile [11.52 °C] to its 95th percentile [24.45 °C] while controlling for all covariates. All hazard ratios are relative to $PM_{2.5}$ at its minimum level [0.82 $\mu g/m^3$]and T_{avg} at its median level [18.14 °C]. All models were adjusted for year and month of conception, weekdays vs. weekends /holidays, maternal age, parity, smoking during pregnancy, area level socio-economic status of the mother and exposure to short- and longer-term gestational (exposure across the entire pregnancy except for the last week before delivery) T_{avg} and longer-term gestational $PM_{2.5}$.

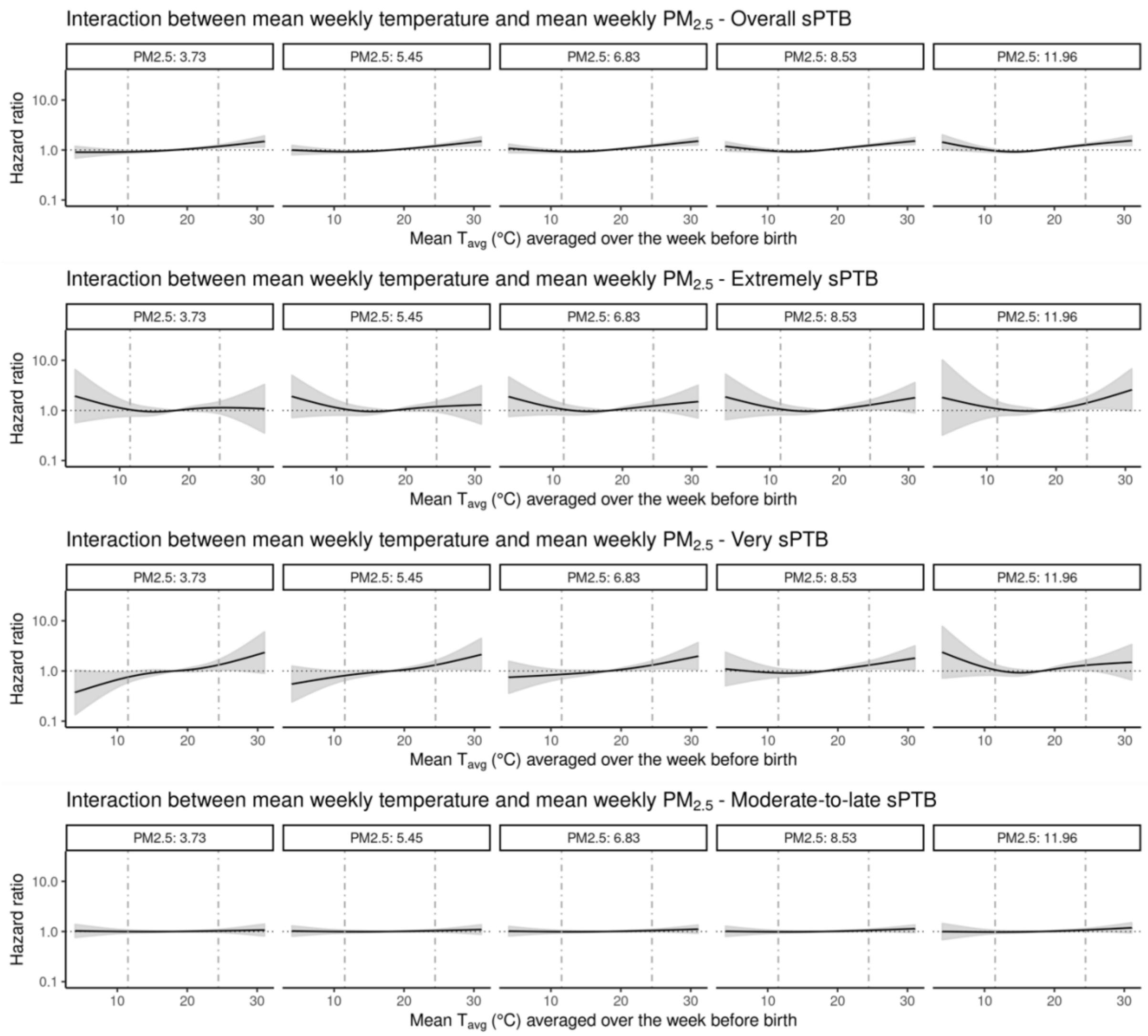

Figure S7: Spontaneous preterm birth (sPTB) hazard ratios (HR) for the interaction between mean $PM_{2.5}$ and mean T_{avg} in the week before delivery derived from the Cox proportional hazard model. The relationship between $PM_{2.5}$ and sPTB was modelled as linear and for T_{avg} as natural cubic spline with three degrees of freedom. The interaction was assessed by introducing a product term between these two variables. The figure shows how the HR change for the association between T_{avg} and sPTB as PM2.5 increases from its 5th percentile (3.73 µg/m^3) to its 95th percentile (11.96 µg/m^3) while controlling for all covariates. All hazard ratios are relative to $PM_{2.5}$ at its median level [6.83 µg/m^3] and T_{avg} at its median level [18.14 °C]. All models were adjusted for year and month of conception, weekdays vs. weekends /holidays, maternal age, parity, smoking during pregnancy, area level socio-economic status of the mother and exposure to short- and longer-term gestational (exposure across the entire pregnancy except for the last week before delivery) T_{avg} and longer-term gestational $PM_{2.5}$.

Table S5: Hazard ratios and confidence intervals for the short-term exposure to 4-week mean $PM_{2.5}$ (in the last four weeks before delivery) in the 4-week exposure model for all spontaneous preterm birth (sPTB) groups in the Greater Metropolitan Region of New South Wales between 1 January 2001 and 31 December 2019

sPTB group	HR for $PM_{2.5}$ per 10 µg/m^3
**Overall**	0.906 (0.876,0.937)
**Extremely**	0.743 (0.624,0.885)
**Very**	0.854 (0.752,0.971)
**Moderate-to-late**	0.969 (0.936,1.003)

Note: Hazard ratios (HR) are shown for 10 µg/m^3 increase in 4-week $PM_{2.5}$. All Cox proportional hazard models were adjusted for year and month of conception, weekdays vs. weekends /holidays, maternal age, parity, smoking during pregnancy, area level socio-economic status of the mother and exposure to short-term and longer-term gestational 4-week $PM_{2.5}$ and 4-week T_{avg}. Short- and longer-term gestational 4 week-T_{avg} were entered as a natural cubic spline with three degrees of freedom in the model and 4-week $PM_{2.5}$ as linear term. Longer-term gestational exposure includes exposure throughout the entire pregnancy except of the last four weeks before delivery.

Table S6: Hazard ratios and confidence intervals for the short-term exposure to 4-week mean temperature (T_{avg}) (in the last four weeks before delivery) in the 4-week exposure model for all spontaneous preterm birth (sPTB) groups in the Greater Metropolitan Region of New South Wales between 1 January 2001 and 31 December 2019

	**Temperature percentiles (°C)**			
**sPTB group**	**5th** (11.9)	**25th** (14.1)	**75th** (21.6)	**95th** (24.0)
Overall	0.84 (0.784,0.9)	0.839 (0.798,0.883)	1.25 (1.2,1.302)	1.427 (1.339,1.521)
Extremely	1.079 (0.779,1.495)	1.022 (0.809,1.292)	1.093 (0.926,1.291)	1.235 (0.945,1.614)
Very	0.654 (0.505,0.847)	0.691 (0.573,0.832)	1.377 (1.196,1.587)	1.551 (1.241,1.94)
Moderate-to-late	0.954 (0.884,1.028)	0.951 (0.901,1.003)	1.071 (1.024,1.119)	1.116 (1.041,1.196)

Note: All Cox proportional hazard models were adjusted for year and month of conception, weekdays vs. weekends /holidays, maternal age, parity, smoking during pregnancy, area level socio-economic status of the mother and exposure to short-term and longer-term gestational 4-week $PM_{2.5}$ and 4-week T_{avg}. Short- and longer-term gestational 4 week-T_{avg} were entered as a natural cubic spline with three degrees of freedom in the model and 4-week $PM_{2.5}$ as linear term. Longer-term gestational exposure includes exposure throughout the entire pregnancy except of the last four weeks before delivery. All hazard ratio estimates are relative to the median 4-weekly mean temperature (18.2°C).

Table S7: Spontaneous Preterm (sPTB) birth hazard ratios and 95% confidence intervals for the interaction effect of 4-week T_{avg} on 4-week $PM_{2.5}$ in the last four gestational weeks before delivery for all live births in the Greater Metropolitan Region of New South Wales between 1 January 2001 and 31 December 2019.

		**$PM_{2.5}$ percentile** (μg/m^3)			
**sPTB group**	**Temperature percentile**	5th (4.6)	25th (6.0)	75th (8.2)	95th (10.9)
Overall sPTB	5th	0.958 (0.932, 0.984)	0.934 (0.894, 0.975)	0.896 (0.836,0.961)	0.854 (0.773, 0.944)
	95th	0.986 (0.963, 1.01)	0.978 (0.942, 1.016)	0.965 (0.908,1.025)	0.95 (0.871, 1.036)
Extremely sPTB	5th	1.007 (0.89, 1.139)	1.01 (0.832, 1.228)	1.017 (0.744,1.389)	1.024 (0.654, 1.603)
	95th	1.006 (0.908, 1.114)	1.009 (0.858, 1.186)	1.014 (0.782,1.315)	1.021 (0.703, 1.482)
Very sPTB	5th	1.01 (0.926, 1.101)	1.015 (0.885, 1.165)	1.025 (0.823,1.276)	1.036 (0.756, 1.42)
	95th	0.933 (0.861, 1.011)	0.896 (0.789, 1.017)	0.838 (0.684,1.027)	0.776 (0.58, 1.039)
Moderate-to-late sPTB	5th	0.973 (0.945, 1.002)	0.958 (0.914, 1.003)	0.933 (0.866,1.005)	0.905 (0.813, 1.007)
	95th	1.01 (0.985, 1.036)	1.016 (0.976, 1.057)	1.026 (0.962,1.093)	1.037 (0.946, 1.137)

Note: The interaction was assessed in a Cox proportional hazard model by introducing a product term between 4-week T_{avg} and 4-week $PM_{2.5}$. The effect of temperature on $PM_{2.5}$ is shown at low (5th percentile = 11.9 °C) and high (95th percentile = 25.5 °C) temperature levels. Hazard ratios are compared to the lowest value of 4-week $PM_{2.5}$ = 2.2 μg/m^3 with 4-week T_{avg} at its median level (18.2 °C). All

models were adjusted for year and month of conception, weekdays vs. weekends /holidays, maternal age, parity, smoking during pregnancy, area level socio-economic status of the mother and exposure to longer-term (i.e., exposure across the entire pregnancy except for in the last four weeks before delivery) gestational 4-week T_{avg} and 4-week $PM_{2.5}$. The relationship between short-term and longer-term gestational 4-week $PM_{2.5}$ and sPTB was modelled as linear and for short-term and longer-term gestational 4-week T_{avg} as natural cubic splines with three degrees of freedom.

Table S8: Spontaneous Preterm (sPTB) birth hazard ratios and 95% confidence intervals for the interaction effect of 4-week $PM_{2.5}$ on 4-week T_{avg} in the last four gestational weeks before delivery for all live births in the Greater Metropolitan Region of New South Wales between 1 January 2001 and 31 December 2019.

		Temperature percentile (°C)				
**sPTB group**	**PM2.5 percentile**	5th (11.9)	25th (14.1)	50th (18.2)	75th (21.6)	95th (24.0)
Overall sPTB	5th	0.981 (0.95, 1.013)	1.014 (1.003, 1.025)	1.212 (1.135,1.294)	1.646 (1.492,1.817)	2.21 (1.978, 2.468)
	95th	1.067 (1.023, 1.113)	0.996 (0.982, 1.009)	1.352 (1.266,1.445)	2.024 (1.827,2.243)	2.493 (2.221, 2.798)
Extremely sPTB	5th	1.057 (0.783, 1.428)	1.03 (0.881, 1.205)	1.004 (0.893, 1.129)	1.223 (0.915,1.635)	1.691 (1.151, 2.486)
	95th	1.268 (0.884, 1.818)	1.052 (0.895, 1.237)	1.05 (0.924, 1.193)	1.314 (0.943,1.831)	1.585 (1.046, 2.403)
Very sPTB	5th	1 (0.966, 1.035)	1.041 (0.922, 1.175)	1.49 (1.128, 1.966)	2.269 (1.559,3.306)	2.947 (1.937, 4.483)
	95th	1.004 (0.96, 1.05)	1.017 (0.87, 1.189)	1.354 (1.014, 1.808)	1.878 (1.287,2.742)	2.245 (1.46, 3.452)
Moderate-to-late sPTB	5th	0.989 (0.949, 1.032)	1.002 (0.997, 1.006)	1.049 (0.981, 1.122)	1.161 (1.046,1.288)	1.3 (1.156, 1.462)
	95th	1.032 (0.977, 1.09)	1 (0.994, 1.005)	1.133 (1.059, 1.213)	1.343 (1.203,1.499)	1.473 (1.302, 1.667)

Note: The interaction was assessed in a Cox proportional hazard model by introducing a product term between 4-week T_{avg} and 4-week $PM_{2.5}$. The effect of 4-week $PM_{2.5}$ on 4-week T_{avg} is shown at low (5th percentile = 4.6 μg/m^3) and high (95th percentile = 10.9 μg/m^3) 4-week $PM_{2.5}$ levels. Hazard ratios are

relative to median 4-week T_{avg} [18.2 °C] and median 4-week $PM_{2.5}$ [7.2 $\mu g/m^3$]. All models were adjusted for year and month of conception, weekdays vs. weekends /holidays, maternal age, parity, smoking during pregnancy, area level socio-economic status of the mother and exposure to longer-term (i.e., exposure across the entire pregnancy except for the last four weeks before delivery) gestational 4-week T_{avg} and 4-week $PM_{2.5}$. The relationship between short-term and longer-term gestational 4-week $PM_{2.5}$ and sPTB was modelled as linear and for short-term and longer-term gestational 4-week T_{avg} as natural cubic splines with three degrees of freedom.

Table S9: Hazard ratios and confidence intervals for short-term exposure to $PM_{2.5}$ as a natural cubic spline term for all spontaneous preterm birth (sPTB) groups in the Greater Metropolitan Region of New South Wales between 1 January 2001 and 31 December 2019 for $PM_{2.5.}$

	**$PM_{2.5}$ percentile** (μg/m³)				
**sPTB group**	5th (3.7)	25th (5.5)	50th (6.8)	75th (8.5)	95th (12.0)
Overall sPTB	0.943 (0.89,1)	0.914 (0.84,0.996)	0.89 (0.814,0.983)	0.874 (0.795,0.961)	0.839 (0.766,0.92)
Extremely sPTB	0.854 (0.656,1.114)	0.792 (0.544,1.148)	0.75 (0.497,1.14)	0.721 (0.476,1.091)	0.664 (0.446,0.989)
Very sPTB	0.998 (0.80342,1.24035)	0.987 (0.729,1.339)	0.969 (0.688,1.363)	0.935 (0.664,1.316)	0.868 (0.625,1.207)
Moderate-to-late sPTB	0.966 (0.906,1.03)	0.95 (0.868,1.041)	0.94 (0.85,1.042)	0.935 (0.844,1.035)	0.925 (0.838,1.02)

Note: All Cox proportional hazard models were adjusted for year and month of conception, weekdays vs. weekends /holidays, maternal age, parity, smoking during pregnancy, area level socio-economic status of the mother and exposure to short- (exposure in the last gestational week before delivery) and longer-term gestational (exposure across the entire pregnancy except for the last week before delivery) $PM_{2.5}$ and T_{avg}. Longer-term gestational $PM_{2.5}$ was entered as a linear term in the model, and short-term, longer-term gestational T_{avg} and short-term $PM_{2.5}$ were entered as natural cubic splines with three degrees of freedom. The reference in all models for percentile comparison is the lowest value of $PM_{2.5}$ = 0.82 μg/m³.

Table S10: Hazard ratios for the short-term (in the last gestational week before delivery) exposure to T_{avg} with exposure to $PM_{2.5}$ modelled as a non-linear term for all spontaneous preterm birth (sPTB) groups in the Greater Metropolitan Region of New South Wales between 1 January 2001 and 31 December 2019.

	**Temperature percentile (°C)**			
**sPTB group**	5th (11.5)	25th (14.1)	75th (21.6)	95th (24.5)
Overall sPTB	0.939 (0.89,0.992)	0.935 (0.902,0.97)	1.112 (1.079,1.147)	1.234 (1.173,1.297)
Extremely sPTB	1.072 (0.831,1.383)	0.985 (0.829,1.17)	1.12 (0.985,1.274)	1.273 (1.026,1.581)
Very sPTB	0.866 (0.706,1.062)	0.907 (0.791,1.04)	1.122 (1.004,1.253)	1.269 (1.059,1.521)
Moderate-to-late sPTB	0.991 (0.934,1.051)	0.99 (0.952,1.029)	1.025 (0.992,1.06)	1.06 (1.004,1.119)

Note: All Cox proportional hazard models were adjusted for year and month of conception, weekdays vs. weekends /holidays, maternal age, parity, smoking during pregnancy, area level socio-economic status of the mother and exposure to short ((i.e., exposure during last gestational week before birth) and longer-term gestational $PM_{2.5}$ (i.e., exposure across the entire pregnancy except for the last week) and longer-term gestational T_{avg}. Longer-term gestational $PM_{2.5}$ was entered as a linear term in the model, and short-term, longer-term gestational T_{avg} and short-term $PM_{2.5}$ were entered as natural cubic splines with three degrees of freedom.. All hazard ratio estimates are relative to the median weekly mean temperature (18.1°C).

Table S11: Spontaneous preterm birth (sPTB) hazard ratios and 95% confidence intervals for the interaction effect between mean T_{avg} and mean $PM_{2.5}$, with $PM_{2.5}$ as a non-linear term in the week before delivery for all live births in the Greater Metropolitan Region of New South Wales between 1 January 2001 and 31 December 2019.

		**$PM_{2.5}$ percentile** (µg/m^3)			
**sPTB group**	**Temperature percentile**	5th (3.7)	25th (5.5)	75th (8.5)	95th (12.0)
Overall PTB	5th	0.771 (0.69, 0.861)	0.694 (0.596, 0.809)	0.679 (0.578,0.797)	0.699 (0.603, 0.81)
	95th	1.349 (1.061, 1.716)	1.526 (1.082, 2.152)	1.587 (1.065,2.364)	1.497 (1.016, 2.207)
Extremely PTB	5th	0.907 (0.55, 1.494)	0.873 (0.439, 1.736)	0.862 (0.427,1.741)	0.76 (0.395, 1.463)
	95th	1.104 (0.39, 3.13)	1.158 (0.262, 5.108)	1.214 (0.219,6.724)	1.221 (0.232, 6.421)
Very PTB	5th	0.628 (0.417, 0.947)	0.534 (0.303, 0.94)	0.572 (0.319,1.025)	0.677 (0.396, 1.158)
	95th	1.214 (0.499, 2.955)	1.299 (0.367, 4.604)	1.26 (0.295,5.384)	1.118 (0.273, 4.578)
Moderate-to-late PTB	5th	0.827 (0.734, 0.932)	0.766 (0.649, 0.904)	0.748 (0.629,0.89)	0.763 (0.65, 0.896)
	95th	1.25 (0.965, 1.619)	1.375 (0.95, 1.99)	1.443 (0.94,2.216)	1.419 (0.935, 2.156)

Note: The interaction was assessed in a Cox proportional hazard model by introducing a product term between T_{avg} and $PM_{2.5}$. The effect of temperature on $PM_{2.5}$ is shown at low (5th percentile = 11.5 °C) and high (95th percentile = 24.5 °C) temperature levels. Hazard ratios are compared to the lowest value

of $PM_{2.5}$ [0.8 μg/m^3] with T_{avg} at its median level [18.2 °C]. All models were adjusted for year and month of conception, weekdays vs. weekends /holidays, maternal age, parity, smoking during pregnancy, area level socio-economic status of the mother and exposure to longer-term (i.e., exposure across the entire pregnancy except for the last week before delivery) gestational T_{avg} and $PM_{2.5}$. Longer-term gestational $PM_{2.5}$ was entered as a linear term in the model, and short-term, longer-term gestational T_{avg} and short-term $PM_{2.5}$ were entered as natural cubic splines with three degrees of freedom.

Table S12: Spontaneous Preterm (sPTB) birth hazard ratios and 95% confidence intervals for the interaction effect of $PM_{2.5}$ as non-linear term on T_{avg} for all live births in the Greater Metropolitan Region of New South Wales between 1 January 2001 and 31 December 2019.

		Temperature percentile (°C)			
**sPTB subcategory**	**$PM_{2.5}$ percentile**	**5th** (11.5 °C)	**25th** (14.1 °C)	**75th** (21.6 °C)	**95th** (24.5°C)
Overall PTB	5th	0.907 (0.849, 0.969)	0.923 (0.881, 0.967)	1.075 (1.035,1.116)	1.109 (1.005, 1.223)
	95th	1.068 (0.956, 1.194)	0.969 (0.918, 1.023)	1.137 (1.089,1.188)	1.279 (1.196, 1.367)
Extremely PTB	5th	0.97 (0.702, 1.329)	0.872 (0.692, 1.098)	1.089 (0.901,1.316)	0.93 (0.537, 1.606)
	95th	1.18 (0.773, 1.794)	1.021 (0.808, 1.291)	1.178 (0.955,1.454)	1.47 (1.073, 2.001)
Very PTB	5th	0.78 (0.599, 1.013)	0.861 (0.71, 1.044)	1.12 (0.959,1.312)	1.22 (0.78, 1.913)
	95th	1.28 (0.94, 1.736)	1.034 (0.864, 1.238)	1.18 (1.008,1.38)	1.41 (1.101, 1.801)
Moderate-to-late PTB	5th	0.981 (0.915, 1.052)	0.985 (0.939, 1.033)	1.004 (0.967,1.043)	0.99 (0.905, 1.084)
	95th	1.043 (0.907, 1.2)	1.002 (0.941, 1.067)	1.035 (0.988,1.085)	1.071 (0.997, 1.15)

Note: The interaction was assessed in a Cox proportional hazard model by introducing a product term between short-term exposure to T_{avg} and $PM_{2.5}$. Short-term T_{avg} and short-term $PM_{2.5}$ were entered as a natural cubic spline with three degrees of freedom. All hazard ratios are relative to $PM_{2.5}$ at its median level [6.83 μg/m^3] and T_{avg} at its median level [18.14 °C]. All models were adjusted for year and month of

conception, weekdays vs. weekends /holidays, maternal age, parity, smoking during pregnancy, area level socio-economic status of the mother and exposure to short- (the week before delivery) and longer-term gestational (exposure across the entire pregnancy except for the last week before delivery) T_{avg} and longer-term gestational $PM_{2.5}$. Longer-term gestational $PM_{2.5}$ was entered as a linear term in the model, and short-term, longer-term gestational T_{avg} and short-term $PM_{2.5}$ were entered as natural cubic splines with three degrees of freedom.
